# What Do Younger and Well-Educated Adults Think about Self-Medication? Results of a Survey during a Public Science Event at Leipzig University

**DOI:** 10.3390/pharmacy12050131

**Published:** 2024-08-23

**Authors:** Ines Gebert, Sabine Hundertmark, Thilo Bertsche

**Affiliations:** 1 Clinical Pharmacy, Institute of Pharmacy, Medical Faculty, Leipzig University, Bruederstraße 32, 04103 Leipzig, Germany; ines.gebert@uni-leipzig.de (I.G.);; 2 Drug Safety Center, Leipzig University, Leipzig University Hospital, Bruederstraße 32, 04103 Leipzig, Germany

**Keywords:** self-medication, self-care, pharmacy, survey

## Abstract

Background: Consecutive visitors to a public science event at Leipzig University were asked about their opinions/attitudes regarding their personal use of self-medication. Methods: A written questionnaire survey addressed (i) participants’ characteristics, (ii) frequency of self-medication use in the last 12 months, (iii) symptoms/complaints most frequently considered applicable, (iv) preconditions, (v) limitations, (vi) risks, (vii) fears, (viii) medication information sources, (ix) influencing factors, and (x) reasons for decision making. Results: (i) A total of 189 visitors (median age: 29.0 years; Q25/Q75: 22.0/44.0) participated, of whom 64.0% were female, 38.6% had a university degree, 20.1% were in training, and 14.8% were licensed in a healthcare profession. (ii) A total of 59.3% of participants stated that they had used self-medication regularly in the last 12 months. The most common answers in the respective questions were (iii) headache, 86.2%; (iv) mild complaints/symptoms, 94.7%; (v) duration, 91.6%; (vi) “self-medication may cause adverse drug reactions”, 94.2%; (vii) “developing a habituation effect”, 58.7%; (viii) pharmacists, 93.7%; (ix) “physician’s recommendation”, 89.3%; (x) “intensity of complaints”, 92.6%; and (vi) 61.3% believed that they could choose an appropriate self-medication. Conclusion: Younger and well-educated adults report using self-medication frequently and rate their expertise as high. Healthcare professionals are the preferred source of information.

## 1. Introduction

Self-medication is a part of self-care and is defined as the patient taking medication independent of a physician’s prescription to increase their well-being [1]. Generally, over-the-counter (OTC) products are used for this purpose, although products other than medications, such as dietary supplements, functional foods, and homeopathy, may also be involved [2,3].

Self-medication saves statutory and private health insurance costs for medications since unnecessary consultations with the general practitioner (GP) can be prevented, as well as emergency department visits [1]. Follow-up costs and losses in productivity can also be avoided by using OTC products when appropriate. The costs paid by patients for self-medication are usually comparatively moderate compared to Rx (prescription medication) products [1]. In terms of revenue, self-medication accounts for 6.2% of sales in community pharmacies in Germany, which corresponded to EUR 3,960,000 in 2022. In terms of items sold, this amounts to 503,000,000 items and 35.8% of all OTC products [4]. The 7-day prevalence of OTC use reached 52.0% for women and 40.8% for men [5]. Self-medication has shown an increasing trend in recent years [4]. Many switches from Rx products to the OTC market have further increased the importance of self-medication in recent years, for instance, the “morning-after pill” [6].

Depending on the country, OTC products may also be available outside the pharmacy or can be ordered online. The local pharmacy, however, has a special role in providing advice on this topic. In Germany, pharmacies are the main dispensaries that offer medications. Dietary supplements and functional foods are also available outside pharmacies, but only some specific medications. In the area of self-medication, the pharmacy is often not only the first but also the only point of contact to check whether self-medication is appropriate according to the patient’s self-diagnosis or whether the limits of self-medication have been reached. Whether and when the limit for self-medication is reached depends, among other things, on the symptoms and complaints, their duration and severity, as well as concurrent conditions and existing medication. To decide which self-medication is best suited to the patient’s needs and to weigh the benefits against the risks, a pharmacist should be able to advise the patient using appropriate communication skills [7]. The pharmacist as a healthcare professional with a university education and a practical background can evaluate important information from the patient [8]. Last but not least, the risk of self-medication [9] can be minimized through expert advice in the pharmacy by identifying contraindications and interactions, and pointing out measures for the prevention or handling of adverse drug reactions (e.g., recommending an appointment with a physician).

The pharmacy is a particularly low-threshold point of contact, offering reliable pharmaceutical advice provided by the pharmaceutical staff, including pharmacy technicians [10], without needing an appointment. Additionally, the emergency service is also available for self-medication around the clock. In Germany, pharmaceutical staff (including pharmacists, pharmacy engineers, and pharmaceutical technical employees) may provide advice on medications, such as self-medication. In Germany, as in all other European Union states, pharmacists are qualified to dispense and advise on all types of medications, including self-medication, after completing their scientific studies at a university and subsequent practical training in a pharmacy (at least half of this practical training is to be completed in a community pharmacy). No additional specialization is mandatory for this professional field. The situation is similar for the other pharmaceutical professionals (e.g., pharmaceutical technical employees and pharmacy engineers), who similarly receive their knowledge of self-medication and adequate customer counseling as part of the corresponding training structures. Additionally, a very limited number of non-pharmacy medications can be sold outside the pharmacy. A “qualified person” must be present on site for these.

In the global literature, many studies have examined aspects of self-medication. Interestingly, the use of analgesics in the area of self-medication, for example, was significantly correlated with a younger age group and the level of education [11]. Also, other authors reported a focus on younger people with regards to self-medication [12]. Typical complaints in studies from abroad dealing with self-medication most frequently included the common cold, headache, fever, and cough [13]. Nevertheless, the direct transferability of such international studies to Western healthcare systems should be considered with caution. One reason for this is that reimbursement of medical costs varies greatly, and, in addition, the general population in developing countries is often significantly younger in principle. Studies from Europe also show that a focus on younger people, such as adolescents, in the context of self-medication is worthwhile [14]. According to those studies, around half of this age group take self-medication, and the assessment of benefits and risks in younger as well as in older multimorbid patients is definitely an issue [14]. So far, other studies addressing self-medication in Germany have mainly focused on studies in the community pharmacy setting [15,16]. The pharmaceutical staff’s view of self-medication and possible sources of information have also already been investigated [17].

However, since the decision to use a product for self-medication lies primarily with the patient, not only should they be included in the considerations, but the measures should be aimed directly at them. Despite this requirement, the question of how younger, well-educated potential users of self-medication view the situation has not yet been adequately addressed in the scientific literature. The typical pharmacy customer tends to belong to the middle/older age group, for whom existing illnesses and medication frequently have to be considered in addition to their current complaints [18]. Younger, well-educated potential self-medication users might not only be expected to be found in pharmacies, but could also be more likely to use online sources. This is why the opinions and views of this group are of particular interest if we want to win them back to the local pharmacy. We, therefore, deliberately aimed to reach a scientifically interested young audience that is not usually the focus of such studies since they do not seem to be the typical customers. It is often claimed that younger people are more likely to get their information online as well as buy medications online—especially self-medication.

For this reason, we made a conscious decision in this study to conduct a survey of potential users on the opportunities and risks of self-medication as part of a popular university science event. We were primarily looking for younger, well-educated people. We deliberately opted for a setting outside the pharmacy to minimize the risk of influence and expectations during the survey. We acted as a university institute and did not present ourselves as pharmacists.

In this project, we aimed to close the presented gap in literature by focusing on younger people interested in science. We invited them to participate in a questionnaire survey during a public science event. This event took place at Leipzig University as an open day event organized by the university every two years. During the event, researchers at Leipzig University give unique insight into their research work and research life. We aimed to investigate attitudes, experiences, and expectations about self-medication among a collective of well-educated young adults.

## 2. Materials and Methods

### 2.1. Participants, Inclusion Criteria, Study Design, and Setting

We consecutively invited all adult visitors of the public science event at Leipzig University on 23 June 2023 to voluntarily participate in our survey. The event is held every two years, and it offers the public the opportunity to gain small insights into the various university research fields and projects. Inclusion criteria were age ≥18 years, sufficient language level to understand the questions asked in the questionnaire, and having not yet participated in the survey. There were no additional exclusion criteria. The survey was carried out prospectively, using a questionnaire about opinions and experiences with self-medication. The survey was set up at our department’s information stand in the entrance area of the lecture theater building, where lectures were being held as part of the public science event. The participants could choose whether to fill out the questionnaire close to the information stand or to take the questionnaire with them and return the filled-out questionnaire later that evening. By submitting their questionnaire, participants were invited to participate in a raffle in which a backpack and small items from the university shop were raffled off. A ticket number without a name was used for this purpose.

### 2.2. Conception of the Questionnaire

For the survey, we created a questionnaire (Supplementary Materials S1) that was forwarded to the participants in written form. It consisted of questions regarding the opinions in several categories addressing self-medication.

The beginning of the questionnaire contained a written informed consent and data protection declaration. The survey was designed to take about 10 minutes to fill out. Ten questions from five main categories (i.e., frequency of use, appropriate indications for self-medication, information-seeking behavior, limits and risks of self-medication, and decision-making factors) were chosen for the questionnaire to address user behavior and attitudes towards self-medication.

All questions, except “frequency of use” and “appropriate indications for self-medication”, were presented as a main question with several subcategories, each to be assessed separately. Each subcategory was to be rated by the respondents using a four-point Likert scale in the context of the main question. Each Likert scale contained two agreement and two disagreement rating categories (strongly agree, partially agree, partially disagree, or strongly disagree). The question “frequency of use” was designed as a single-choice question, as was the question “appropriate indications for self-medication”, but with the difference that each reply counted for each indication (appropriate/not appropriate) on its own.

In addition, a free text category was offered for most questions to add categories and aspects that had not yet been listed.

The ten main questions covered the following topics (in categories iii-x) were as follows: participants’ characteristics (i), frequency of self-medication use (ii), symptoms/complaints considered appropriate for self-medication (iii), preconditions for self-medication use (iv), limitations for self-medication use (v), risks in self-medication use (vi), fears in self-medication use (vii), sources of information on medications for self-medication use (viii), influencing factors for self-medication use (ix), and reasons for decision making for self-medication use (x). The questionnaire was pretested in two rounds. In the first round, the survey was pretested amongst ten people with no healthcare profession background or affiliation to the project. In the second round, the survey was pretested by pharmacists experienced in self-medication practice in community pharmacies. The socio-demographic characteristics (category i) were asked at the end of the questionnaire. All other categories were presented in the order shown here.

### 2.3. Preprocessing, Digitalization, and Analysis

The returned questionnaires were digitized using the software FormPro (by OCR Systeme GmbH, Leipzig, Germany, Version: 3.1, 2020). Furthermore, the digitized data were compared with the corresponding paper questionnaires, and it was checked whether all data were digitalized correctly. Once all questionnaires were correctly digitized, a dataset for Excel was created. The Excel (by Microsoft, Redmond, WA; USA, Version: Excel 2405; operating system: Windows) dataset also formed the basis for the SPSS (by IBM, Armonk, NY, USA, SPSS Inc.; operating system: Windows, Version: SPSS Statistics 29) dataset. SPSS and Excel were subsequently used for visualization and descriptive data analysis. The dataset is available on request from the authors.

## 3. Results

### 3.1. Participants’ Characteristics (i)

In total, 189 visitors participated in our survey. The median age of all participants was 29.0 years (Q25: 22.0/Q75: 44.0), with an age range from 18.0 to 71.0 years of age. A total of 64.0% of the participants identified as female, 30.7% as male, and 5.3% as diverse or did not wish to disclose their gender. When asked for the highest educational qualification, 38.6% held a university degree certificate, 29.1% were undergraduates at the current moment, and 15.3% had completed vocational training. The remaining participants reported that they either were in vocational training (4.2%), had a school-leaving certificate without a higher professional degree (8.4%), or were pupils (1.1%) at the time. A total of 57.1% of the survey participants had no affiliation with a health profession, while 20.1% were in active training and 14.8% were professionally licensed in a health profession. We show the results of the participants’ reports depending on gender and health profession in Supplementary Materials S2.

### 3.2. Frequency of Self-Medication Use (ii)

**Figure 1:**
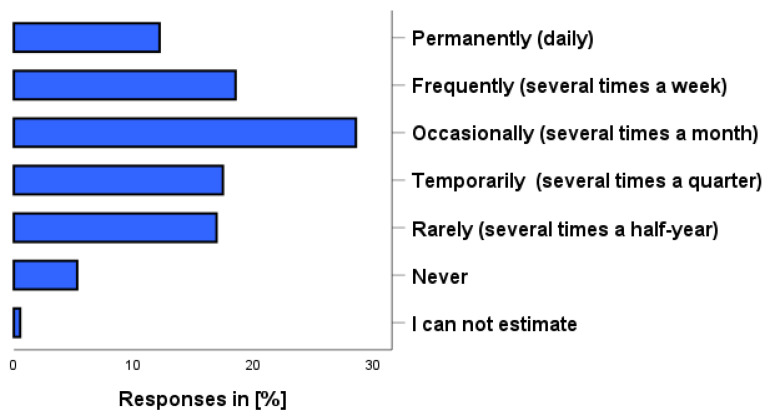
How often have you used self-medication preparations in the past 12 months? Please also think about dietary supplements and herbal preparations. Please just one answer.

As shown in Figure 1, participants reported using self-medication within the last 12 months, including dietary supplements, daily (12.2%), several times a week (18.5%), several times a month (28.6%), several times a quarter (17.5%), or several times in a half-year (16.9%). A total of 5.3% reported that they had not used any self-medication within the last 12 months and 0.5% could not estimate their use.

### 3.3. Symptoms/Complaints Considered Appropriate for Self-Medication (iii)

**Figure 2:**
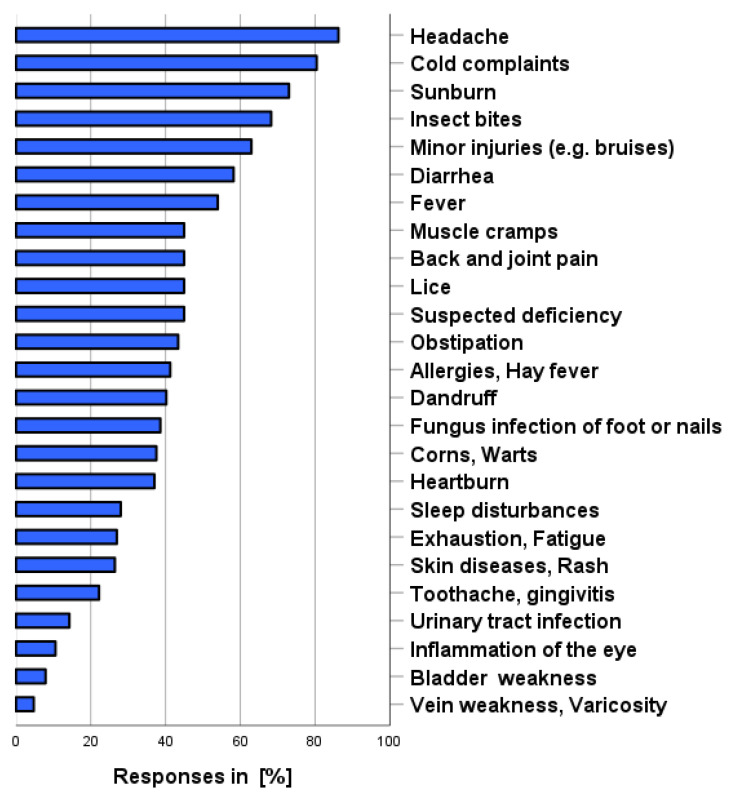
For which symptoms/complaints would you resort to self-medication before seeing a physician? Multiple answers are possible.

As shown in Figure 2, the following symptoms/complaints were most frequently considered to apply to the use of self-medication (own use, categories possible and before consulting a physician): headache (86.2%), cold complaints (80.4%), sunburn (73.0%), insect bites (68.3%), minor injuries such as bruises/cuts (63.0%), and diarrhea (58.2%). Female participants were asked to rate three additional complaints, resulting in the following frequencies: menstrual complaints (43.4%), use of emergency contraception (18.0%), and menopausal complaints (9.5%). Venous insufficiency/varicose veins (4.8%), bladder weakness (7.9%), inflammation of the eye (10.6%), and urinary tract infections were rated as the symptoms/complaints least likely to be treated with OTC products.

### 3.4. Preconditions for Self-Medication Use (iv)

**Figure 3:**
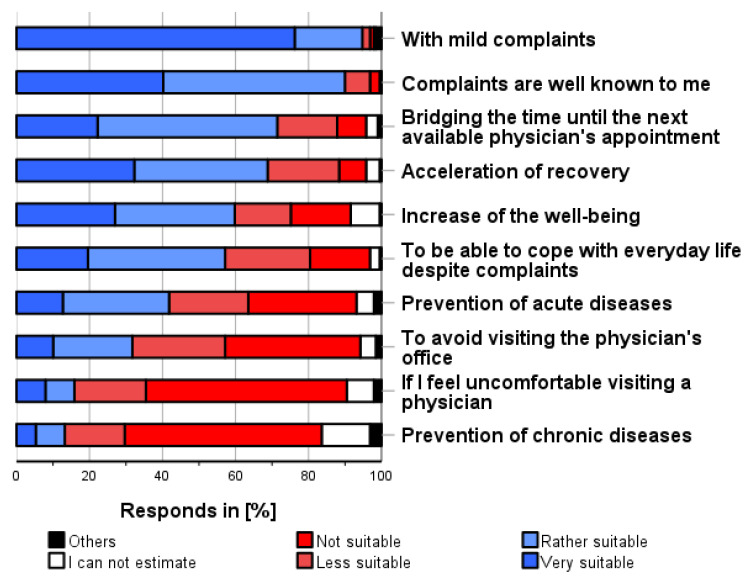
In which situations do you think it is appropriate to use self-medication before consulting a physician? Please only one answer option per line.

As shown in Figure 3, the following conditions were most frequently considered applicable for self-medication use (independent of personal use, multiple categories possible and before consulting a physician): “Mild complaints/symptoms” (94.7%), “Well-known complaints/symptoms” (90.0%), “Bridging the time until the next available physician’s appointment” (71.4%), and “Expectation of speeding up their recovery” (68.8%).

Using self-medication for the prevention of chronic diseases (13.2%), because one is uncomfortable seeing a physician (15.9%), or to avoid visiting a physician’s office (31.7%), were considered as the least appropriate situations for self-medication.

### 3.5. Limitations for Self-Medication Use (v)

**Figure 4:**
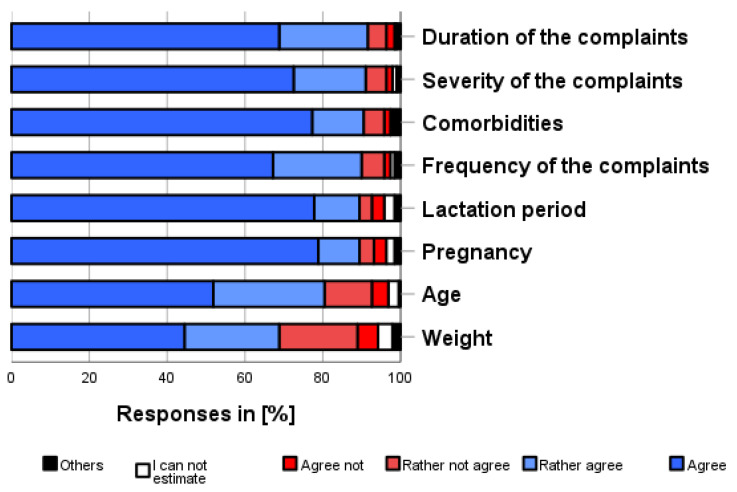
How much do you agree with the following aspects? Please only one answer option per line. Whether a self-medication product is appropriate for a person depends on.

As shown in Figure 4, the following limitations were most frequently considered applicable for self-medication use (independent of own use, multiple categories possible and before consulting a physician): duration (91.6%), severity of the complaints (91.0%), comorbidities (90.4%), and frequency of complaints (90.0%). The least important limitations were age (80.5%) and weight (68.7%).

### 3.6. Risks in Self-Medication Use (vi)

**Figure 5:**
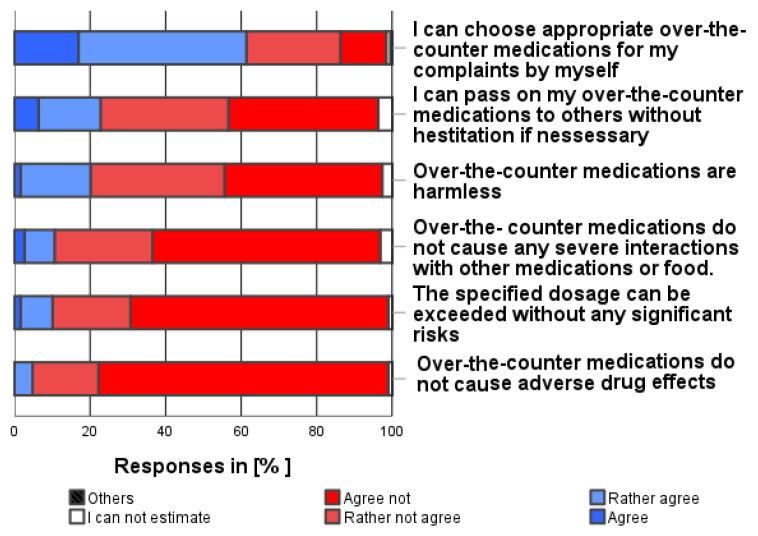
How much do you agree with the following statements about the safety of over-the-counter medications in general? Please only one answer option per line.

A total of 61.3% of the participants were convinced that they could choose an appropriate over-the-counter medication for their complaints/symptoms on their own (see Figure 5). As shown in Figure 5, the following safety risks were most frequently associated with self-medication use (independent of own use, multiple categories possible and before consulting a physician):

”Self-medication may cause adverse drug reactions” (94.2%), “The specified dosages can be exceeded without any significant risk.” (88.9%), “Self-medication may cause severe drug interactions” (86.2%), “Self-medication is not harmless” (77.6%), and “Self-medication cannot easily be passed on to others” (73.6%).

### 3.7. Fears about Self-Medication Use (vii)

**Figure 6:**
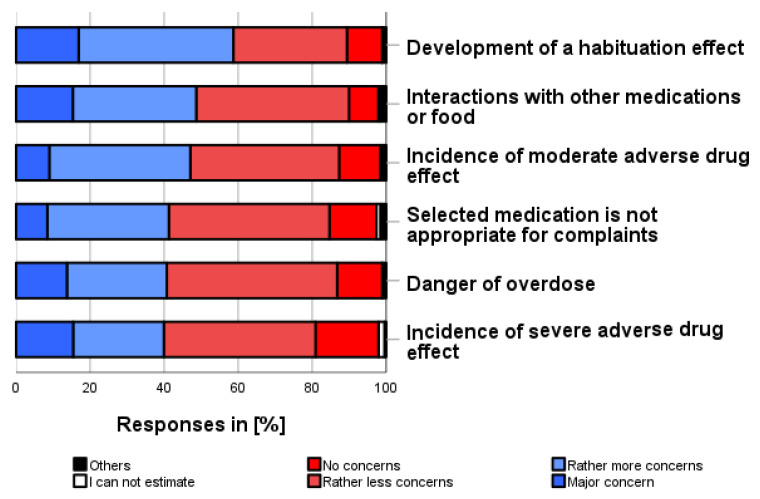
What fears do you have about self-medication? Please only one answer option per line.

As shown in Figure 6, the following fears were the most common that the participants had concerning the use of self-medication (independent of own use, multiple categories possible and before consulting a physician): “Developing a habituation effect” (58.7%), “Interactions with other medication or food” (48.6%) and “Occurrence of moderate adverse drug reactions” (47.1%). In addition, 41.3% feared that the selected medication was not appropriate for their symptom/complaint, 40.8% feared that they might overdose their self-medication, and 39.9% feared that severe adverse drug reactions might occur.

### 3.8. Sources of Information on Medications for Self-Medication Use (viii)

**Figure 7:**
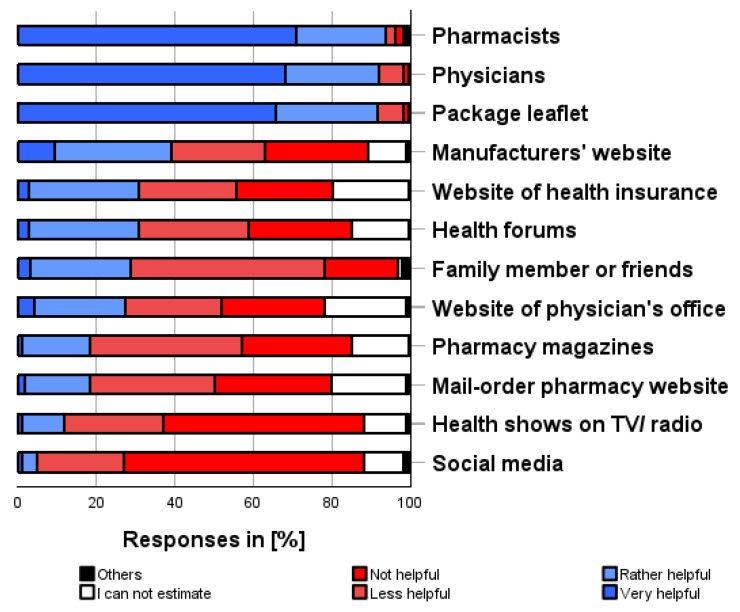
How helpful do you think the following sources of information are for finding out about OTC products? Please only one answer option per line.

As shown in Figure 7, the following sources of information on medications for self-medication were most helpful in obtaining information about self-medication (multiple categories possible): pharmacists (93.7%), physicians (92.1%), package leaflets (91.5%), manufacturers’ website (39.1%). In contrast, social media (83.6%), health shows on TV/radio (76.7%), mail-order pharmacy websites (61.3%), and pharmacy magazines (66.6%) were most frequently reported as sources of information on medications that were not or were less helpful in obtaining valid information about self-medication preparations.

**Figure 8:**
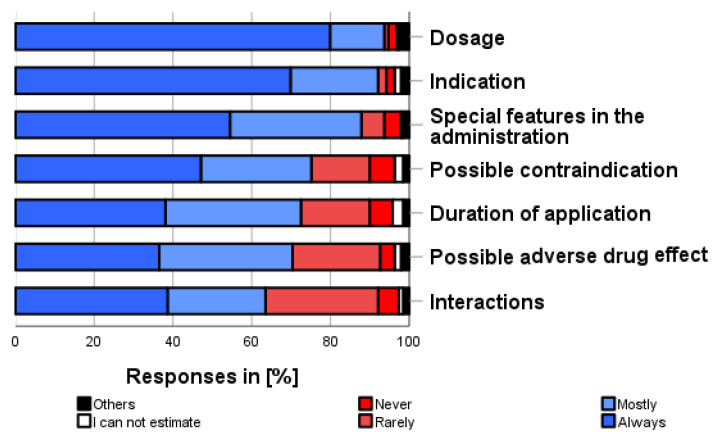
How often do you inform yourself about the following aspects when using self-medication for the first time? Please only one answer option per line.

The most frequently obtained information (see Figure 8) about self-medication preparation, before using them for the first time, was about dosage (93.7%), indication (92.0%), administration and medication handling (87.8%), contraindication (75.1%), duration of use (72.5%), adverse drug reactions (70.4%), and interaction with other medications (63.5%).

### 3.9. Factors Influencing the Use of Self-Medication (ix)

**Figure 9:**
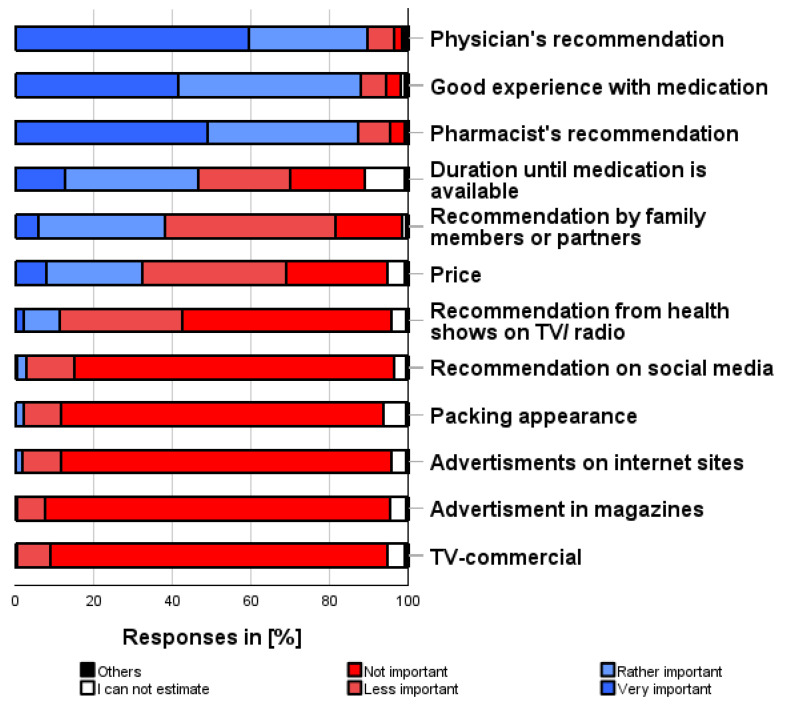
How important are the following factors to you when deciding to self-medicate? Please only one answer option per line.

As shown in Figure 9, the following influencing factors were most frequently taken into account when deciding whether to use a self-medication preparation at all (multiple categories possible):

“Physician’s recommendation” (89.5%), “Good personal experience” (87.9%), “Pharmacist’s recommendation” (87.3%), “Duration until the prescribed medication is available” (46.6%), and “Price” (32.2%).

“TV commercials” (94.2%), “Advertisements in magazines” (94.7%), or “Advertisements on websites” (94.2%) were the three factors reported to be the least important in the decision-making process.

### 3.10. Reasons for Decision to Use Self-Medication (x)

**Figure 10:**
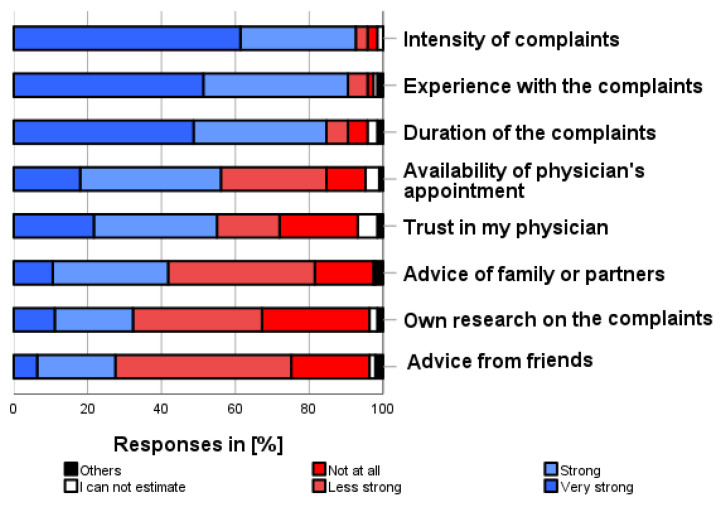
How much do the following factors influence your decision to self-medicate before seeing a physician? Please only one answer option per line.

As shown in Figure 10, the following factors were the most important reasons for deciding to use (a particular) self-medication or to consult a physician (several categories possible): “Intensity of complaints” (92.6%), “Experience with the complaints” (90.5%), “Duration of the complaints” (84.7%), “Availability of a physician’s appointment” (56.1%), and “Trust in physician” (55.0%).

## 4. Discussion

### 4.1. General Considerations and Principal Findings

Self-medication has become increasingly relevant in recent years. In addition to external evidence and a pharmacist’s experience, knowing the patient’s wishes and expectations is a central component of evidence-based pharmacy [19]. This is because, in contrast to the reimbursement of prescriptions by healthcare insurance, patients (as the customers) generally have to pay for self-medication themselves. As the costs of self-medication are not shared by a solidarity system, it is up to patients to decide which services they are willing to pay for and at what price. In addition, in the case of self-medication, the pharmaceutical industry mainly addresses its advertising directly to the consumer. All this underlines the need to look at self-medication from the patient’s point of view. To this end, we wanted to invite all visitors to participate in a questionnaire survey during the public science event, whereby we expected a high proportion of younger visitors with an academic background.

### 4.2. Methodological Aspects: Self-Medication—Younger People as a Target Group

It is often assumed that mainly older patients self-medicate and seek advice in pharmacies [20]. However, many elderly patients have pre-existing conditions that require careful consideration of the complaints, as more severe symptoms or adverse drug reactions may result from the existing therapy. The limits of self-medication are then quickly reached [18]. The same applies to children and adolescents, who are a particularly vulnerable group and should be seen by a pediatrician at an early stage if they suffer from complaints, especially when the complaints occur for the first time [21].

However, younger adults also have complaints and illnesses that are particularly amenable to self-medication. Therefore, the perspective of younger adults—even those without chronic illnesses—should be of particular interest in this regard, as addressed in our survey.

Several studies have elucidated the importance of self-medication in younger, well-educated adults worldwide [22,23,24]. However, the assessment of self-medication also depends on the country’s health system. Data from Europe and Germany that take a critical view of self-medication from independent institutions such as universities are comparatively rare.

In a survey conducted by the KBV (National Association of Statutory Health Insurance Physicians) in 2019, 31% of the interviewees up to age 29 and 42% of those aged 29–39 stated that they had problems finding a GP in their area [25]. Although the same interviewees reported that 94% and 93% of these age groups, respectively, currently have a GP, the problem of shortages of GPs will become significantly more critical in the future because 36.9% of GPs are already over 60 years old [26]. This makes the local pharmacy all the more important as the first point of contact for health issues.

An additional argument for investigating self-medication behavior at a younger age is that preventive approaches to maintaining health through self-medication and dietary supplements should be started at a younger age and require an appropriate attitude. It is also claimed that younger adults, the target group of our survey, are particularly critical of sources of information such as pharmacies and are more likely to obtain information from the Internet. Younger adults are said to underestimate the risks of self-medication and to order self-medication uncritically on the Internet. We wanted to get to the bottom of this.

### 4.3. Methodological Aspects: Types of Self-Medication

The appropriateness and limits of each indication for self-medication should be defined individually, considering existing concomitant complaints, the duration and severity of the current symptoms, pre-existing conditions, and current medication. The main focus of self-medication is the treatment of acute, short-term, self-terminating disorders that do not require a visit to the physician. Self-medication may also be indicated for a limited time for longer-lasting complaints. In this case, however, it is essential to consult a physician if the symptoms persist or worsen. Furthermore, self-medication can also be used as a stopgap measure if a visit to the physician is advisable and medical treatment is indicated. In such a case, however, it is essential to point out that self-medication aims to bridge the gap in the short term. In principle, self-medication can be considered with and without chronic underlying illnesses. Of course, self-medication should be carefully coordinated with any long-term therapy or underlying diseases. Contraindications and medication interactions should be considered, particularly in such cases. Another situation is when a patient receives a medically prescribed treatment, but now requests an over-the-counter continuation, intermediate therapy, or an alternative from the pharmacy. Even if such a situation can be justified in individual cases, such as triptan therapy [27] in self-medication with a physician’s diagnosis, it generally seems doubtful whether self-medication, possibly without the physician’s knowledge, is sensible if treatment is prescribed at the same time. Simultaneous intake of similar or identical products can quickly lead to overdoses.

### 4.4. Aspects of Content: Frequent Use and Benefits of Self-Medication

We managed to attract a respectable number of 189 participants; almost everyone who came to our stand was persuaded to participate. As expected, with a median age of 29, the participants were significantly younger than the usual pharmacy customers. The majority of participants were female, many had a university degree, and a significant number worked in the healthcare sector. We did not expect to find a high number of regular self-medication users, more precisely, people who had self-medicated at least once in the last 12 months. However, an overwhelming majority of almost 94% reported this, with many reporting regular use. The focus of their self-medication was on minor health problems in particular, which—especially in the absence of chronic illnesses, as is often the case with younger people—do not justify a visit to the physician, at least if the health problem is temporary. The survey showed that respondents were aware of the conditions under which self-medication is justified. Mild, known complaints and bridging the gap until a visit to the physician were consequently the focus of their self-medication treatment. It should be noted that we did not ask whether the respondents had self-medicated for the indications featured in the questionnaire, e.g., headache, cold complaints, and sunburn. However, this seems very likely.

### 4.5. Aspects of Content: Limitations and Risks of Self-Medication

This is also consistent with the fact that the respondents considered the limited duration, the severity of the complaints, and possible comorbidities, in particular, to be factors where self-medication may no longer be justified. On the other hand, the respondents were also well aware that self-medication can be associated with risks. The overwhelming rejection of the admittedly provocative questions about the risks shows that the participants responded very consciously and did not simply agree blindly with our statements. “Self-medication may cause adverse drug reactions”, “Self-medication may cause severe interaction with medications, “and “Exceeding the recommended dosage can lead to risks in self-medication” were the most mentioned risks. In line with these findings, participants also expressed fears about self-medication; “Developing a habituation effect”, “Interactions with other medication or food”, and “Occurrence of moderate adverse drug reactions” were the most common fears. However, it should be noted that correct use, especially for a limited time, and the opportunity to consult with pharmacists can help reduce fears and ensure adherence, and thus treatment success.

### 4.6. Aspects of Content: Decision-Making Aids for Self-Medication

The preferred sources of information were somewhat surprising. While pharmacists, physicians, and package leaflets were consistently cited as the most reliable source of information on medications for self-medication, unspecified internet sources, social media, and internet forums were rated at the bottom of the list. This is surprising, as one would expect these sources to be relevant among younger people. The fact that traditional sources and knowledgeable healthcare professionals were mentioned in the first place should incentivize them to continue this task in the future. This is also underlined by the fact that “Physician’s recommendation”, “Pharmacist’s recommendation”, and “Good own experience” were ultimately the most frequently cited factors influencing the specific decision to self-medicate. It is important to note that the survey was conducted by a neutral university institution, not by a pharmacy or physician’s practice. Ultimately, however, the following parameters were most often the deciding factors for self-medication: “Intensity of complaints”, “Experience with the complaints”, and “Duration of the complaints”.

### 4.7. Indicators for Future Considerations

The survey also revealed contradictions that justify a consultation at the pharmacy despite the good level of information. For example, over 61% stated that they could choose an appropriate preparation for their complaints themselves. On the other hand, 41.3% were concerned that the selected preparation was not appropriate for their complaints. In addition, around 60% had relatively little or no concern about overdosing with self-medication; previously, however, around 90% stated that exceeding the recommended dosage was not without risk.

### 4.8. Comparison to the Literature Dealing with Self-Medication in a Similar Population

It is interesting to compare our results with a study [28] investigating the knowledge and use of self-medication among medical and pharmacy students. In that study, pharmacy students and female participants were found to have greater knowledge. However, similar results were obtained for the indication group. Again, headache was the most common indication. In [28], more than half (64%) of the students reported using self-medication in the previous 6 months, which was numerically lower than in our survey, which reported a use rate of almost 94% in the last year.

A systematic review and meta-analysis [22] reported that the overall prevalence of self-medication among university students was between these two figures, at 70%. In addition, female students were more likely to self-medicate than male students (odds ratio 1.45). The authors showed a higher prevalence of self-medication in medical students (97%) than in non-medical students (45%) in 60,938 evaluated students [22]. This contrasts with our results, which show no differences in the healthcare groups despite their high proportion in our survey. This difference can be explained simply by the significantly higher number in a meta-analysis. In another study [29], the authors, who included a broader population group than we did, found that 63% had practiced self-medication in the previous three months. The most commonly used medications were painkillers (61%), similar to our survey. This was followed by antibiotics (32%), which are largely unavailable as OTC products in our healthcare system, demonstrating the differences in healthcare systems between countries. Furthermore, the reputation of the pharmacist, who was the source of self-medication for 54%, was similar to our study [29].

Urban and rural areas have already been shown to influence self-medication in terms of health control and the physical, psychological, and spiritual symptoms experienced by a group of students [30].

These results show that transferring results from one region or country to another is challenging and that local surveys are justified to contribute to the assessment, even if some factors are robust and appear independent of the setting.

It can be expected that the level of education and socioeconomic status have a significant influence on assessments and risk evaluations in general and, thus, also in the area of health issues. It has been shown that socioeconomic factors influence the way people with type 2 diabetes change their dietary habits after diagnosis [31]. In addition, the family plays a crucial role in the self-management of diet. Although there are few data on the influence of socioeconomic aspects on self-medication, such an influence can be expected. However, our study focused primarily on younger, well-educated test subjects. Unfortunately, it was therefore not possible to make a sophisticated comparison between different educational groups. Our study was too biased towards well-educated people, and others were underrepresented compared to the “normal” population.

One particular aspect of our study is that, due to the setting, many people who worked in healthcare professions themselves or were in training to do so took part. Even though we found no indications that their views differed fundamentally from those of people outside the healthcare professions, this group was too small for a detailed evaluation within our study. This aspect should therefore be carefully considered while interpreting our data.

### 4.9. Implications for the Future

The results strengthen our resolve to keep an eye on the issue of self-medication among younger people. Even well-educated customers expect good and well-founded care in the local pharmacy. Based on the results of our study, corresponding information services that enable pharmacies to quickly implement evidence-based information about self-medication into pharmacy practice should be expanded and scientifically investigated for their appropriateness to improve patient-related outcomes. Lastly, a better focus on younger adults in self-medication is relevant for the individual patient. It also makes it possible to save medical treatment costs and conserve resources through appropriate self-medication where the patient is referred to a physician in good time if necessary.

### 4.10. Limitations

The present survey has several limitations.

First, it should be recognized that we enrolled a highly selective cohort within our setting. However, this cohort was deliberately selected to fill a research gap among younger, well-educated adults. Therefore, this survey does not claim to represent the general population. Additionally, those working in the medical professions or training were over-represented compared with the “normal” population.

Second, the results reflect only the opinions of the respondents. It is conceivable that some participants gave answers they hoped we would like to receive. The subjective ratings should, therefore, not be misunderstood as objective evaluations. Nevertheless, they do reflect the views of an important user group.

Third, we only asked for general assessments. Of course, depending on the indication or the specific active ingredient, a detailed assessment is required, as not all OTC products can be assessed as a whole. The fact that dietary supplements and products could not be further identified by the participants and were subsequently categorized as self-medication underlines this limitation when considering our results.

Fourth, as with most surveys, recall bias is to be expected.

Fifth, it should be noted that for some questions, we did not ask about actual illnesses and personal health-related background, such as medical history, for data protection reasons. Therefore, it remains open in individual answers whether an assessment was made based on specific personal experience or in general terms.

Sixth, it should be borne in mind that some aspects of self-medication are regulated differently in different countries. Therefore, our results cannot be transferred uncritically to other healthcare systems in which, for example, products that are subject to the pharmacy’s obligation in one country are freely available for sale in another.

Seventh, it should be considered that although the questionnaire was carefully developed by experts in a multi-stage process, including pretesting, it was not validated against any outcomes.

Eighth, the following confounding and bias factors exist. Age or gender can be a confounder. There could also be bias in the results, in particular, from a recall bias, i.e., there are memory gaps when a questionnaire asks respondents to report on previous experiences with self-medication. We saw only a slight bias concerning participants’ expectations, as we acted as a neutral, knowledgeable institution and not as a pharmacy or pharmaceutical company.

## 5. Conclusions

This study shows that younger, well-educated adults are a significant important “target group” for self-medication in the pharmacy. This is the case since the majority of those participants stated that they used self-medication mostly regularly in the last year. However, more than half believed that they could choose a suitable self-medication for themselves. Further, headaches, cold symptoms, and sunburn were mentioned as the most frequent complaints considered suitable for self-medication. The category “Mild complaints/symptoms” was reported as the top precondition, whereas duration was the most frequent limitation. These results also underline a realistic and appropriate assessment of the possibilities and limitations of self-medication in this population. Concerning potential risks, almost all respondents reported that “Self-medication may cause adverse drug reactions”, which underlines a critical point of view of our participants. However, more than half feared a “habituation effect”, which indicates a need for counseling to address unjustified fears. Interestingly, pharmacists, physicians, and package leaflets were the most frequently mentioned medication information sources, while internet sources—surprisingly for this group—were well behind.

***Implications of findings on practice***

1. Younger, well-educated adults are a relevant target group for self-medication.
2. Younger, well-educated adults highly appreciate the counseling services offered by pharmaceutical professionals.
3. Pharmacies should pay more attention to younger, well-educated adults, also considering pre-existing conditions and polymedication.
4. Special product offers for younger, well-educated adults can help to increase their attractiveness when visiting the pharmacy.
5. The pharmaceutical industry should also keep younger, well-educated adults in mind as pharmacy customers.

## Data Availability

The dataset is available on request from the authors with a jointly agreed study concept.

## Supplementary Materials

The following supporting information can be downloaded at: https://www.mdpi.com/article/10.3390/pharmacy12050131/s1, Supplement S1: The drug quiz—Questionnaire (anonymous)-; Supplement S2: Gender and affiliation with a health care profession.

## Abbreviations

GP	General practitioner
KBV	National Association of Statutory Health Insurance Physicians (Kassenaerztliche Bundesvereinigung)
OTC	Over-the-counter
RX	Prescription medication
