# Supplementary material for: What Do Younger and Well-Educated Adults Think about Self-Medication? Results of a Survey during a Public Science Event at Leipzig University
Source: Pharmacy (Basel). 2024 Aug 23;12(5):131. doi: 10.3390/pharmacy12050131 (PMC11417743; doi:10.3390/pharmacy12050131)
Supplement: Supplementary file 1 [file pharmacy-12-00131-s001.zip › Supplement S1_Questionaire.pdf]

UNIVERSITÄT LEIPZIG
Medizinische Fakultät

Universitätsklinikum Leipzig
Medizin ist unsere Berufung.

ZAMS ZENTRUM FÜR ARZNEIMITTELSICHERHEIT

THE DRUG QUIZ

- Questionnaire (anonymous)-

Have you ever thought about the benefits of over-the-counter medicines and whether there are any risks associated with their use? Your opinion is important to us! **By taking part, you can be a part of science yourself.**

THANK YOU VERY MUCH FOR TAKING PART!

Information on participation and data protection

We want to use the answers from this questionnaire as part of a research project to investigate opinions and views on application situations, safety and limits of self-medication. Self-medication refers to the treatment of illnesses with **over-the-counter medicines**, dietary supplements or medical products without consulting a physician first. Participation is of course **voluntary**. This questionnaire will be distributed at the Long Night of Science on 23.06.2023. If you have already taken part in this survey, please do not participate again. By completing the survey, you agree to the anonymous scientific evaluation of your data.

You can contact the data protection officer of the Faculty of Medicine at Leipzig University at Philipp-Rosenthal-Straße 27, Haus M, 04103 Leipzig, e-mail: dsbmf@medizin.uni-leipzig.de. You have the right to lodge a complaint with the Saxon Data Protection Officer (Devrientstraße 5, 01067 Dresden, e-mail: post@sdtb.sachsen.de). We will be happy to answer any questions you may have. Please feel free to contact us at klinische.pharmazie@uni-leipzig.de.

**What is Self-medication?**
Self-medication refers to the independent use of over-the-counter medicines or other over-the-counter healthcare products for the treatment or prevention of illnesses without medical consultation. Other health products are, for example, dietary supplements (e.g. vitamins, minerals) or medical products.

1. **How often have you used self-medication preparations in the past 12 months? Please also think about dietary supplements and herbal preparations.** Please just one answer**.**

☐	Permanently (daily)
☐	Frequently (several times a week)
☐	Occasionally (several times a month)
☐	Temporarily (several times a quarter)
☐	Rarely (several times every six months)
☐	Never
☐	I can not estimate.

2. **For which symptoms/complaints would you resort to self-medication before seeing a doctor? -** Multiple answers are possible**.**

☐	Headache
☐	Allergies, Hay fever
☐	Suspected deficiency
☐	Minor injuries such as bruises abrasions
☐	Back and joint pain
☐	Muscle cramps
☐	Toothache, gingivitis
☐	Inflammation of the eye
☐	Menstrual cramps (women only)
☐	Menopausal symptoms (women only)
☐	Emergency contraception (morning after pill)
☐	Bladder weakness
☐	Urinary tract infection
☐	Heartburn
☐	Diarrhea
☐	Obstipation
☐	Cold complaints
☐	Fever
☐	Sleep disturbances
☐	Exhaustion, Fatigue
☐	Corns, warts
☐	Insect bites
☐	Sunburn
☐	Skin diseases, Rash
☐	Dandruff
☐	Lice
☐	Fungus infection of foot or nails
☐	Vein weakness, varicosity
☐	Other complaints:

3. In which situations do you think it is appropriate to use self-medication before consulting a physician? Please only one answer option per line**.**

	**Very appropriate**	**Rather appropriate**	**Less appropriate**	**Not appropriate**	**I can not estimate**
With mild complaints	□	□	□	□	□
Acceleration of recovery	□	□	□	□	□
To be able to cope with everyday life despite complaints	□	□	□	□	□
Prevention of acute diseases	□	□	□	□	□
Prevention of chronic disease	□	□	□	□	□
Complaints are well known to me	□	□	□	□	□
Increase of the well-being	□	□	□	□	□
If I feel uncomfortable visiting a physician	□	□	□	□	□
To avoid visiting the physician's office	□	□	□	□	□
Bridging the time until the next available physician's appointment	□	□	□	□	□
Other situation: …………………………………………	□	□	□	□	□

4. How much do you agree with the following aspects? Please only one answer option per line.
Whether a self-medication product is appropriate for a person depends on:

	**Agree**	**Rather agree**	**Rather not agree**	**I do not agree**	**I can not estimate**
Age	□	□	□	□	□
Weight	□	□	□	□	□
Comorbidities	□	□	□	□	□
Severity of the complaints	□	□	□	□	□
Duration of the complaints	□	□	□	□	□
Frequency of the complaints	□	□	□	□	□
Pregnancy	□	□	□	□	□
Lactation period	□	□	□	□	□
Others:	□	□	□	□	□

5. **How much do you agree with the following statements about the safety of over-the-counter medications in general?** Please only one answer option per line.

	**Agree**	**Rather agree**	**Rather not agree**	**I do not agree**	**I can not estimate**
Over-the-counter medications are harmless.	◻	◻	◻	◻	◻
Over-the-counter medications do not cause adverse drug effects	◻	◻	◻	◻	◻
Over-the-counter medications do not cause any severe interactions with other medications or foods.	◻	◻	◻	◻	◻
The specified dosages can be exceeded without any significant risks.	◻	◻	◻	◻	◻
I can choose appropriate over-the counter medications for my complaints by myself.	◻	◻	◻	◻	◻
I can pass on my over-the-counter medications to others (e.g. a friend or colleague) without hesitation if necessary.	◻	◻	◻	◻	◻

6. **What fears do you have about self-medication?** Please only one answer option per line

	**Major concerns**	**Rather more concerns**	**Rather less concerns**	**No concerns**	**I can not estimate**
Development of a habituation effect	◻	◻	◻	◻	◻
Danger of overdose	◻	◻	◻	◻	◻
Incidence of moderate adverse drug effect	◻	◻	◻	◻	◻
Incidence of severe adverse drug effect	◻	◻	◻	◻	◻
Selected medication is not appropriate for complaints	◻	◻	◻	◻	◻
Interactions with other medication or food	◻	◻	◻	◻	◻
Others:	◻	◻	◻	◻	◻

7. **How helpful do you think the following sources of information are for finding out about self-medication products?** Please only one answer option per line.

	**Very helpful**	**Rather helpful**	**Less helpful**	**Not helpful**	**I can not estimate**
Package leaflet	□	□	□	□	□
Physicians	□	□	□	□	□
Pharmacists	□	□	□	□	□
Family member or friends	□	□	□	□	□
Pharmacy magazines	□	□	□	□	□
Health shows on TV/ radio	□	□	□	□	□
Social media	□	□	□	□	□
Manufacturers' website	□	□	□	□	□
Health forums	□	□	□	□	□
Websites of health insurance	□	□	□	□	□
Mail-order pharmacy websites	□	□	□	□	□
Website of physician's office	□	□	□	□	□
Others:	□	□	□	□	□

8. **How often do you inform yourself about the following aspects when using self-medication <u>for the first time</u>?** Please only one answer option per line.

	**Always**	**Mostly**		**Rarely**	**Never**	**I can not estimate**
Indication	□	□		□	□	□
Dosage	□	□		□	□	□
Special features in the administration	□	□		□	□	□
Possible adverse drug effect	□	□		□	□	□
Interactions	□	□		□	□	□
Maximum duration of use without medical advice	□	□		□	□	□
Possible contraindication	□	□		□	□	□

9. **How important are the following factors to you when deciding to self-medicate?** Please only one answer option per line.

	Very important	Rather important	Less important	Not Important	I can not estimate
Physician's recommendation	☐	☐	☐	☐	☐
Pharmacist's recommendation	☐	☐	☐	☐	☐
Recommendation by family members or partners	☐	☐	☐	☐	☐
Recommendation from health shows on TV/ radio	☐	☐	☐	☐	☐
Good experience with medication	☐	☐	☐	☐	☐
Price	☐	☐	☐	☐	☐
Duration until medication is available	☐	☐	☐	☐	☐
Packing appearance	☐	☐	☐	☐	☐
TV-commercial	☐	☐	☐	☐	☐
Advertising in magazines	☐	☐	☐	☐	☐
Advertisements on internet sites	☐	☐	☐	☐	☐
Recommendation on social media	☐	☐	☐	☐	☐
Other factors: ……………………………………………………………	☐	☐	☐	☐	☐

10. **How much do the following factors influence your decision to self-medicate before seeing a physician?** Please only one answer option per line**.**

	Very strong	Strong	Less strong	Not at all	I can not estimate
Intensity of complaints	☐	☐	☐	☐	☐
Duration of the complaints	☐	☐	☐	☐	☐
Experience with the complaints	☐	☐	☐	☐	☐
Advice of family or partners	☐	☐	☐	☐	☐
Advice from friends	☐	☐	☐	☐	☐
Own research on the complaints	☐	☐	☐	☐	☐
Availability of physician's appointment	☐	☐	☐	☐	☐
Trust in my physician	☐	☐	☐	☐	☐
Other factors: …………………………………………………………	☐	☐	☐	☐	☐

Please give us a few more details about yourself. This information is of course voluntary and will help us to assess the representativeness of the results.

Age: ______ Years

Sex: □ Female □ male □ divers or no specification

School-leaving certificate:

□ Without a general school-leaving certificate
□ Still in school
□ Secondary school leaving certificate (Haupt-(Volks-)schulabschluss)
□ Certificate o the polytechnischen Oberschule (POS)
□ Secondary school leaving certificate (Realschul- oder gleichwertiger Abschluss)
□ University entrance qualification (Fachhochschul- oder Hochschulreife (Abitur))

Educational attainment:

□ Without further professional education
□ Still in vocational training
□ completed vocational training
□ Still in studies
□ University degree (Bachelor, Master, Diplom, Staatsexamen, or similar)

Are you currently training for a healthcare profession?
□ I am training to be: **…………………………………….**
□ I study: ……………………………………………………
□ No

Do you belong to a healthcare profession?
□Yes, I am a/an**: ………………………………………**
□ No
