# Supplementary material for: What Do Younger and Well-Educated Adults Think about Self-Medication? Results of a Survey during a Public Science Event at Leipzig University
Source: Pharmacy (Basel). 2024 Aug 23;12(5):131. doi: 10.3390/pharmacy12050131 (PMC11417743; doi:10.3390/pharmacy12050131)
Supplement: Supplementary file 1 [file pharmacy-12-00131-s001.zip › Supplement S2_Replies per subgroup_Gender_Affiliation.pdf]

1. **How often have you used self-medication preparations in the past 12 months? Please also think about dietary supplements and herbal preparations**. Please just one answer.	Gender (n =189)						Affiliation with a healthcare profession (n= 189)					
	Female (n = 121)		Male (= 58)		Non-binary or no specified (n=10)		Others (N/A) (n = 15)		Affiliated (fully trained & in training) (n=66)		No affiliation (n = 108)	
	absolut	%	absolut	%	absolut	%	absolut	%	absolut	%	absolut	%
Participants replies - Main question follow by it's subcategories; broken down by gender and affiliation to a healthcare profession												
Permanently (daily)	15	12.4	6	10.3	2	20.0	1	6.7	5	7.6	17	15.7
Frequently (several times a week)	20	16.5	13	22.4	2	20.0	6	40.0	13	19.7	16	14.8
Occasionally (several times a month)	40	33.1	11	19.0	3	30.0	3	20.0	26	39.4	25	23.1
Temporarily (several times a quarter)	21	17.4	12	20.7	0	0.0	0	0.0	12	18.2	21	19.4
Rarely (several times every six months)	18	14.9	12	20.7	2	20.0	3	20.0	8	12.1	21	19.4
Never	6	5.0	4	6.9	0	0.0	2	13.3	2	3.0	6	5.6
I can not estimate.	0	0.0	0	0.0	1	10.0	0	0.0	0	0.0	1	0.9
Others (N/A)	1	0.8	0	0.0	0	0.0	0	0.0	0	0.0	1	0.9

2. **For which symptoms/complaints would you resort to self-medication before seeing a doctor?** - Multiple answers are possible. (Table only shows results of the reply "appropriate indication")

Participants replies - Main question follow by it's subcategories; broken down by gender and affiliation to a healthcare profession												
Headache	105	86.8	51	87.9	7	70.0	13	86.7	59	89.4	91	84.3
Allergies, Hay fever	48	39.7	26	44.8	4	40.0	4	26.7	35	53.0	39	36.1
Suspected deficiency	53	43.8	27	46.6	5	50.0	6	40.0	31	47.0	48	44.4
Minor injuries such as bruises abrasions	83	68.6	33	56.9	3	30.0	9	60.0	37	56.1	73	67.6
Back and joint pain	61	50.4	21	36.2	3	30.0	6	40.0	26	39.4	53	49.1
Muscle cramps	56	46.3	25	43.1	4	40.0	1	6.7	35	53.0	49	45.4
Toothache, gingivitis	29	24.0	11	19.0	2	20.0	3	20.0	15	22.7	24	22.2
Inflammation of the eye	11	9.1	8	13.8	1	10.0	1	6.7	8	12.1	11	10.2
Menstrual cramps (women only)	78	64.5	0	0.0	4	40.0	4	26.7	38	57.6	40	37.0
Menopausal symptoms (women only)	16	13.2	0	0.0	2	20.0	3	20.0	8	12.1	7	6.5
Emergency contraception (morning after pill)	30	24.8	0	0.0	4	40.0	4	26.7	16	24.2	14	13.0
Bladder weakness	11	9.1	2	3.4	2	20.0	1	6.7	3	4.5	11	10.2
Urinary tract infection	21	17.4	3	5.2	3	30.0	1	6.7	14	21.2	12	11.1
Heartburn	35	28.9	30	51.7	5	50.0	5	33.3	28	42.4	37	34.3
Diarrhea	67	55.4	38	65.5	5	50.0	9	60.0	41	62.1	60	55.6
Obstipation	53	43.8	26	44.8	3	30.0	6	40.0	30	45.5	46	42.6
Cold complaints	97	80.2	47	81.0	8	80.0	10	66.7	56	84.8	86	79.6
Fever	65	53.7	31	53.4	6	60.0	9	60.0	39	59.1	54	50.0
Sleep disturbances	32	26.4	17	29.3	4	40.0	6	40.0	20	30.3	27	25.0
Exhaustion, Fatigue	33	27.3	15	25.9	3	30.0	5	33.3	10	15.2	36	33.3
Corns, warts	51	42.1	18	31.0	2	20.0	2	13.3	28	42.4	41	38.0
Insect bites	84	69.4	39	67.2	6	60.0	9	60.0	45	68.2	75	69.4
Sunburn	90	74.4	42	72.4	6	60.0	10	66.7	51	77.3	77	71.3
Skin diseases, Rash	31	25.6	17	29.3	2	20.0	2	13.3	17	25.8	31	28.7
Dandruff	49	40.5	22	37.9	5	50.0	5	33.3	25	37.9	46	42.6
Lice	57	47.1	23	39.7	5	50.0	8	53.3	29	43.9	48	44.4
Fungus infection of foot or nails	48	39.7	22	37.9	3	30.0	4	26.7	31	47.0	38	35.2
Vein weakness, varicosity	6	5.0	2	3.4	1	10.0	1	6.7	4	6.1	4	3.7

3. In which situations do you think it is appropriate to use self-medication before consulting a physician? Please only one answer option per line.

Participants replies - Main question follow by it's subcategories; broken down by gender and affiliation to a healthcare profession		Gender (n =189)						Affiliation with a healthcare profession (n= 189)					
		Female (n = 121)		Male (= 58)		Non-binary or no specified (n=10)		Others (N/A) (n = 15)		Affiliated (fully trained & in training) (n=66)		No affiliation (n = 108)	
		absolut	%	absolut	%	absolut	%	absolut	%	absolut	%	absolut	%
With mild complaints	**Very suitable**	94	77.7	42	72.4	8	80.0	7	46.7	55	83.3	82	75.9
	**Rather suitable**	20	16.5	14	24.1	1	10.0	6	40.0	9	13.6	20	18.5
	**Less suitable**	3	2.5	1	1.7	0	0.0	1	6.7	0	0.0	3	2.8
	**Not suitable**	1	0.8	0	0.0	1	10.0	0	0.0	1	1.5	1	0.9
	**I can not estimate**	1	0.8	0	0.0	0	0.0	0	0.0	0	0.0	1	0.9
	**Others (N/A)**	2	1.7	0	0.0	0	0.0	1	6.7	1	1.5	1	0.9
Acceleration of recovery	**Very suitable**	36	29.8	19	32.8	6	60.0	4	26.7	24	36.4	33	30.6
	**Rather suitable**	47	38.8	20	34.5	2	20.0	6	40.0	19	28.8	44	40.7
	**Less suitable**	22	18.2	14	24.1	1	10.0	3	20.0	16	24.2	18	16.7
	**Not suitable**	12	9.9	2	3.4	0	0.0	1	6.7	6	9.1	7	6.5
	**I can not estimate**	4	3.3	2	3.4	1	10.0	0	0.0	1	1.5	6	5.6
	**Others (N/A)**	0	0.0	1	1.7	0	0.0	1	6.7	0	0.0	0	0.0
To be able to cope with everyday life despite complaints	**Very suitable**	23	19.0	11	19.0	3	30.0	5	33.3	13	19.7	19	17.6
	**Rather suitable**	49	40.5	18	31.0	4	40.0	2	13.3	24	36.4	45	41.7
	**Less suitable**	29	24.0	14	24.1	1	10.0	3	20.0	20	30.3	21	19.4
	**Not suitable**	17	14.0	12	20.7	2	20.0	2	13.3	9	13.6	20	18.5
	**I can not estimate**	3	2.5	2	3.4	0	0.0	2	13.3	0	0.0	3	2.8
	**Others (N/A)**	0	0.0	1	1.7	0	0.0	1	6.7	0	0.0	0	0.0
Prevention of acute diseases	**Very suitable**	15	12.4	8	13.8	1	10.0	2	13.3	8	12.1	14	13.0
	**Rather suitable**	37	30.6	14	24.1	4	40.0	3	20.0	20	30.3	32	29.6
	**Less suitable**	24	19.8	17	29.3	0	0.0	2	13.3	16	24.2	23	21.3
	**Not suitable**	38	31.4	14	24.1	4	40.0	6	40.0	19	28.8	31	28.7
	**I can not estimate**	5	4.1	4	6.9	0	0.0	1	6.7	0	0.0	8	7.4
	**Others (N/A)**	2	1.7	1	1.7	1	10.0	1	6.7	3	4.5	0	0.0
Prevention of chronic disease	**Very suitable**	4	3.3	6	10.3	0	0.0	1	6.7	4	6.1	5	4.6
	**Rather suitable**	10	8.3	5	8.6	0	0.0	1	6.7	4	6.1	10	9.3
	**Less suitable**	23	19.0	6	10.3	2	20.0	3	20.0	12	18.2	16	14.8
	**Not suitable**	70	57.9	28	48.3	4	40.0	7	46.7	39	59.1	56	51.9
	**I can not estimate**	12	9.9	11	19.0	2	20.0	1	6.7	5	7.6	19	17.6
	**Others (N/A)**	2	1.7	2	3.4	2	20.0	2	13.3	2	3.0	2	1.9
Complaints are well known to me	**Very suitable**	47	38.8	23	39.7	6	60.0	7	46.7	28	42.4	41	38.0
	**Rather suitable**	62	51.2	29	50.0	3	30.0	6	40.0	32	48.5	56	51.9
	**Less suitable**	9	7.4	3	5.2	1	10.0	0	0.0	4	6.1	9	8.3
	**Not suitable**	3	2.5	2	3.4	0	0.0	2	13.3	2	3.0	1	0.9
	**I can not estimate**	0	0.0	1	1.7	0	0.0	0	0.0	0	0.0	1	0.9
	**Others (N/A)**	0	0.0	0	0.0	0	0.0	0	0.0	0	0.0	0	0.0
Increase of the well-being	**Very suitable**	29	24.0	17	29.3	5	50.0	4	26.7	15	22.7	32	29.6
	**Rather suitable**	44	36.4	16	27.6	2	20.0	5	33.3	24	36.4	33	30.6
	**Less suitable**	20	16.5	9	15.5	0	0.0	1	6.7	11	16.7	17	15.7
	**Not suitable**	16	13.2	13	22.4	2	20.0	5	33.3	11	16.7	15	13.9
	**I can not estimate**	11	9.1	3	5.2	1	10.0	0	0.0	5	7.6	10	9.3
	**Others (N/A)**	1	0.8	0	0.0	0	0.0	0	0.0	0	0.0	1	0.9
If I feel uncomfortable visiting a physician	**Very suitable**	5	4.1	7	12.1	3	30.0	4	26.7	2	3.0	9	8.3
	**Rather suitable**	11	9.1	4	6.9	0	0.0	0	0.0	2	3.0	13	12.0
	**Less suitable**	24	19.8	11	19.0	2	20.0	3	20.0	13	19.7	21	19.4
	**Not suitable**	68	56.2	31	53.4	5	50.0	8	53.3	41	62.1	55	50.9
	**I can not estimate**	10	8.3	4	6.9	0	0.0	0	0.0	5	7.6	9	8.3
	**Others (N/A)**	3	2.5	1	1.7	0	0.0	0	0.0	3	4.5	1	0.9
To avoid visiting the physician’s office	**Very suitable**	28	23.1	6	10.3	3	30.0	4	26.7	6	9.1	9	8.3
	**Rather suitable**	33	27.3	12	20.7	1	10.0	0	0.0	7	10.6	34	31.5
	**Less suitable**	41	33.9	14	24.1	1	10.0	3	20.0	24	36.4	21	19.4
	**Not suitable**	6	5.0	25	43.1	4	40.0	8	53.3	26	39.4	36	33.3
	**I can not estimate**	0	0.0	1	1.7	1	10.0	0	0.0	2	3.0	6	5.6
	**Others (N/A)**	3	2.5	0	0.0	0	0.0	0	0.0	1	1.5	2	1.9
Bridging the time until the next available physician’s appointment	**Very suitable**	67	55.4	12	20.7	5	50.0	6	40.0	14	21.2	22	20.4
	**Rather suitable**	18	14.9	24	41.4	2	20.0	5	33.3	31	47.0	57	52.8
	**Less suitable**	6	5.0	12	20.7	1	10.0	2	13.3	15	22.7	14	13.0
	**Not suitable**	4	3.3	7	12.1	2	20.0	2	13.3	4	6.1	9	8.3
	**I can not estimate**	0	0.0	2	3.4	0	0.0	0	0.0	2	3.0	4	3.7
	**Others (N/A)**	1	0.8	1	1.7	0	0.0	0	0.0	0	0.0	2	1.9

4. How much do you agree with the following aspects? Please only one answer option per line. **Whether a self-medication product is appropriate for a person depends on:**

Participants replies - Main question follow by it's subcategories; broken down by gender and affiliation to a healthcare profession

		Gender (n =189)						Affiliation with a healthcare profession (n= 189)					
		Female (n = 121)		Male (= 58)		Non-binary or no specified (n=10)		Others (N/A) (n = 15)		Affiliated (fully trained & in training) (n=66)		No affiliation (n = 108)	
		absolut	%	absolut	%	absolut	%	absolut	%	absolut	%	absolut	%
Age	**Agree**	63	52.1	26	44.8	9	90.0	6	40.0	46	69.7	46	42.6
	**Rather agree**	34	28.1	20	34.5	0	0.0	4	26.7	11	16.7	39	36.1
	**Rather not agree**	16	13.2	7	12.1	0	0.0	2	13.3	7	10.6	14	13.0
	**I do not agree**	5	4.1	3	5.2	0	0.0	2	13.3	2	3.0	4	3.7
	**I can not estimate**	3	2.5	2	3.4	0	0.0	0	0.0	0	0.0	5	4.6
	**Others (N/A)**	0	0.0	0	0.0	1	10.0	1	6.7	0	0.0	0	0.0
Weight	**Agree**	57	47.1	19	32.8	8	80.0	5	33.3	35	53.0	44	40.7
	**Rather agree**	27	22.3	18	31.0	1	10.0	5	33.3	14	21.2	27	25.0
	**Rather not agree**	24	19.8	14	24.1	0	0.0	2	13.3	13	19.7	23	21.3
	**I do not agree**	7	5.8	3	5.2	0	0.0	2	13.3	3	4.5	5	4.6
	**I can not estimate**	4	3.3	3	5.2	0	0.0	0	0.0	1	1.5	6	5.6
	**Others (N/A)**	2	1.7	1	1.7	1	10.0	1	6.7	0	0.0	3	2.8
Comorbidities	**Agree**	99	81.8	38	65.5	9	90.0	11	73.3	57	86.4	78	72.2
	**Rather agree**	14	11.6	11	19.0	0	0.0	1	6.7	5	7.6	19	17.6
	**Rather not agree**	3	2.5	7	12.1	0	0.0	2	13.3	3	4.5	5	4.6
	**I do not agree**	2	1.7	1	1.7	0	0.0	0	0.0	1	1.5	2	1.9
	**I can not estimate**	0	0.0	0	0.0	0	0.0	0	0.0	0	0.0	0	0.0
	**Others (N/A)**	3	2.5	1	1.7	1	10.0	1	6.7	0	0.0	4	3.7
Severity of the complaints	**Agree**	94	77.7	35	60.3	8	80.0	11	73.3	52	78.8	74	68.5
	**Rather agree**	17	14.0	17	29.3	1	10.0	1	6.7	10	15.2	24	22.2
	**Rather not agree**	5	4.1	5	8.6	0	0.0	2	13.3	3	4.5	5	4.6
	**I do not agree**	2	1.7	1	1.7	0	0.0	0	0.0	1	1.5	2	1.9
	**I can not estimate**	2	1.7	0	0.0	0	0.0	0	0.0	0	0.0	2	1.9
	**Others (N/A)**	1	0.8	0	0.0	1	10.0	1	6.7	0	0.0	1	0.9
Duration of the complaints	**Agree**	87	71.9	35	60.3	8	80.0	8	53.3	51	77.3	71	65.7
	**Rather agree**	27	22.3	15	25.9	1	10.0	3	20.0	12	18.2	28	25.9
	**Rather not agree**	3	2.5	6	10.3	0	0.0	2	13.3	3	4.5	4	3.7
	**I do not agree**	2	1.7	2	3.4	0	0.0	1	6.7	0	0.0	3	2.8
	**I can not estimate**	1	0.8	0	0.0	0	0.0	0	0.0	0	0.0	1	0.9
	**Others (N/A)**	1	0.8	0	0.0	1	10.0	1	6.7	0	0.0	1	0.9
Frequency of the complaints	**Agree**	89	73.6	29	50.0	9	90.0	9	60.0	52	78.8	66	61.1
	**Rather agree**	25	20.7	18	31.0	0	0.0	2	13.3	9	13.6	32	29.6
	**Rather not agree**	3	2.5	8	13.8	0	0.0	3	20.0	3	4.5	5	4.6
	**I do not agree**	2	1.7	1	1.7	0	0.0	0	0.0	1	1.5	2	1.9
	**I can not estimate**	1	0.8	1	1.7	0	0.0	0	0.0	0	0.0	2	1.9
	**Others (N/A)**	0	0.0	1	1.7	1	10.0	1	6.7	1	1.5	1	0.9
Pregnancy	**Agree**	101	83.5	39	67.2	9	90.0	11	73.3	58	87.9	80	74.1
	**Rather agree**	12	9.9	8	13.8	0	0.0	0	0.0	3	4.5	17	15.7
	**Rather not agree**	3	2.5	4	6.9	0	0.0	2	13.3	3	4.5	2	1.9
	**I do not agree**	3	2.5	3	5.2	0	0.0	1	6.7	2	3.0	3	2.8
	**I can not estimate**	0	0.0	4	6.9	0	0.0	0	0.0	0	0.0	4	3.7
	**Others (N/A)**	2	1.7	0	0.0	1	10.0	1	6.7	0	0.0	2	1.9
Lactation period	**Agree**	101	83.5	37	63.8	9	90.0	10	66.7	58	87.9	79	73.1
	**Rather agree**	13	10.7	9	15.5	0	0.0	1	6.7	3	4.5	18	16.7
	**Rather not agree**	2	1.7	4	6.9	0	0.0	2	13.3	3	4.5	1	0.9
	**I do not agree**	3	2.5	3	5.2	0	0.0	1	6.7	2	3.0	3	2.8
	**I can not estimate**	0	0.0	5	8.6	0	0.0	0	0.0	0	0.0	5	4.6
	**Others (N/A)**	2	1.7	0	0.0	1	10.0	1	6.7	0	0.0	2	1.9

5. How much do you agree with the following statements about the safety of over-the-counter medications in general? Please only one answer option per line.

Participants replies - Main question follow by it's subcategories; broken down by gender and affiliation to a healthcare profession

		Gender (n =189)						Affiliation with a healthcare profession (n= 189)					
		Female		Male (= 58)		Non-binary or no		Others (N/A) (n =		Affiliated (fully trained & in training)		No affiliation (n = 108)	
		absolut	%	absolut	%	absolut	%	absolut	%	absolut	%	absolut	%
Over-the-counter medications are harmless.	**Agree**	0	0.0	3	5.2	0	0.0	1	6.7	1	1.5	1	0.9
	**Rather agree**	17	14.0	16	27.6	2	20.0	0	0.0	8	12.1	27	25.0
	**Rather not agree**	48	39.7	18	31.0	1	10.0	4	26.7	20	30.3	43	39.8
	**I do not agree**	53	43.8	20	34.5	6	60.0	7	46.7	37	56.1	35	32.4
	**I can not estimate**	3	2.5	1	1.7	1	10.0	3	20.0	0	0.0	2	1.9
	**Others (N/A)**	0	0.0	0	0.0	0	0.0	0	0.0	0	0.0	0	0.0
Over-the-counter medications do not cause adverse drug reactions.	**Agree**	0	0.0	0	0.0	0	0.0	0	0.0	0	0.0	0	0.0
	**Rather agree**	2	1.7	7	12.1	0	0.0	2	13.3	1	1.5	6	5.6
	**Rather not agree**	19	15.7	14	24.1	0	0.0	1	6.7	10	15.2	22	20.4
	**I do not agree**	99	81.8	37	63.8	9	90.0	11	73.3	55	83.3	79	73.1
	**I can not estimate**	1	0.8	0	0.0	1	10.0	1	6.7	0	0.0	1	0.9
	**Others (N/A)**	0	0.0	0	0.0	0	0.0	0	0.0	0	0.0	0	0.0
Over-the-counter medications do not cause severe drug interactions with other medications or foods.	**Agree**	1	0.8	4	6.9	0	0.0	0	0.0	0	0.0	5	4.6
	**Rather agree**	5	4.1	10	17.2	0	0.0	2	13.3	3	4.5	10	9.3
	**Rather not agree**	34	28.1	13	22.4	2	20.0	4	26.7	16	24.2	29	26.9
	**I do not agree**	78	64.5	29	50.0	7	70.0	7	46.7	47	71.2	60	55.6
	**I can not estimate**	3	2.5	2	3.4	1	10.0	2	13.3	0	0.0	4	3.7
	**Others (N/A)**	0	0.0	0	0.0	0	0.0	0	0.0	0	0.0	0	0.0
The specified dosages can be exceeded without any significant risk.	**Agree**	0	0.0	3	5.2	0	0.0	0	0.0	1	1.5	2	1.9
	**Rather agree**	9	7.4	7	12.1	0	0.0	1	6.7	1	1.5	14	13.0
	**Rather not agree**	24	19.8	13	22.4	2	20.0	2	13.3	16	24.2	21	19.4
	**I do not agree**	87	71.9	35	60.3	7	70.0	11	73.3	48	72.7	70	64.8
	**I can not estimate**	1	0.8	0	0.0	1	10.0	1	6.7	0	0.0	1	0.9
	**Others (N/A)**	0	0.0	0	0.0	0	0.0	0	0.0	0	0.0	0	0.0
I can choose suitable over-the counter medications for my complaints by myself.	**Agree**	15	12.4	16	27.6	1	10.0	1	6.7	20	30.3	11	10.2
	**Rather agree**	56	46.3	22	37.9	6	60.0	5	33.3	28	42.4	51	47.2
	**Rather not agree**	36	29.8	11	19.0	0	0.0	5	33.3	16	24.2	26	24.1
	**I do not agree**	12	9.9	9	15.5	2	20.0	3	20.0	2	3.0	18	16.7
	**I can not estimate**	1	0.8	0	0.0	1	10.0	1	6.7	0	0.0	1	0.9
	**Others (N/A)**	1	0.8	0	0.0	0	0.0	0	0.0	0	0.0	1	0.9
I can pass on my over-the-counter medications to others (e.g. friend or colleague) without hesitation if necessary.	**Agree**	3	2.5	8	13.8	1	10.0	0	0.0	5	7.6	7	6.5
	**Rather agree**	15	12.4	16	27.6	0	0.0	1	6.7	10	15.2	20	18.5
	**Rather not agree**	52	43.0	10	17.2	2	20.0	3	20.0	26	39.4	35	32.4
	**I do not agree**	49	40.5	22	37.9	4	40.0	8	53.3	25	37.9	42	38.9
	**I can not estimate**	2	1.7	2	3.4	3	30.0	3	20.0	0	0.0	4	3.7
	**Others (N/A)**	0	0.0	0	0.0	0	0.0	0	0.0	0	0.0	0	0.0

**6. What fears do you have about self-medication?** Please only one answer option per line		Gender (n =189)						Affiliation with a healthcare profession (n= 189)					
		Female (n = 121)		Male (= 58)		Non-binary or no specified (n=10)		Others (N/A) (n = 15)		Affiliated (fully trained & in training) (n=66)		No affiliation (n = 108)	
		absolut	%	absolut	%	absolut	%	absolut	%	absolut	%	absolut	%
Participants replies - Main question follow by it's subcategories; broken down by gender and affiliation to a healthcare profession													
Development of a habituation effect	**Major concerns**	18	14.9	10	17.2	4	40.0	5	33.3	12	18.2	15	13.9
	**Rather more concerns**	51	42.1	25	43.1	3	30.0	8	53.3	26	39.4	45	41.7
	**Rather less concerns**	37	30.6	20	34.5	1	10.0	1	6.7	19	28.8	38	35.2
	**No concerns**	15	12.4	2	3.4	1	10.0	0	0.0	8	12.1	10	9.3
	**I can not estimate**	0	0.0	0	0.0	0	0.0	0	0.0	0	0.0	0	0.0
	**Others (N/A)**	0	0.0	1	1.7	1	10.0	1	6.7	1	1.5	0	0.0
Danger of overdose	**Major concerns**	18	14.9	6	10.3	2	20.0	2	13.3	11	16.7	13	12.0
	**Rather more concerns**	37	30.6	14	24.1	0	0.0	6	40.0	17	25.8	28	25.9
	**Rather less concerns**	49	40.5	32	55.2	6	60.0	5	33.3	27	40.9	55	50.9
	**No concerns**	17	14.0	5	8.6	1	10.0	1	6.7	10	15.2	12	11.1
	**I can not estimate**	0	0.0	0	0.0	0	0.0	0	0.0	0	0.0	0	0.0
	**Others (N/A)**	0	0.0	1	1.7	1	10.0	1	6.7	1	1.5	0	0.0
Incidence of moderate adverse drug effect	**Major concerns**	10	8.3	5	8.6	2	20.0	4	26.7	7	10.6	6	5.6
	**Rather more concerns**	49	40.5	20	34.5	3	30.0	5	33.3	25	37.9	42	38.9
	**Rather less concerns**	53	43.8	22	37.9	1	10.0	4	26.7	25	37.9	47	43.5
	**No concerns**	9	7.4	9	15.5	3	30.0	1	6.7	8	12.1	12	11.1
	**I can not estimate**	0	0.0	1	1.7	0	0.0	0	0.0	0	0.0	1	0.9
	**Others (N/A)**	0	0.0	1	1.7	1	10.0	1	6.7	1	1.5	0	0.0
Incidence of severe adverse drug effect	**Major concerns**	19	15.7	6	10.3	4	40.0	3	20.0	12	18.2	14	13.0
	**Rather more concerns**	29	24.0	17	29.3	0	0.0	4	26.7	18	27.3	24	22.2
	**Rather less concerns**	50	41.3	23	39.7	4	40.0	7	46.7	23	34.8	47	43.5
	**No concerns**	21	17.4	10	17.2	1	10.0	0	0.0	13	19.7	19	17.6
	**I can not estimate**	1	0.8	2	3.4	0	0.0	0	0.0	0	0.0	3	2.8
	**Others (N/A)**	0	0.0	0	0.0	1	10.0	1	6.7	0	0.0	0	0.0
Selected medication is not suitable for complaints	**Major concerns**	12	9.9	3	5.2	1	10.0	4	26.7	4	6.1	8	7.4
	**Rather more concerns**	38	31.4	21	36.2	3	30.0	5	33.3	20	30.3	37	34.3
	**Rather less concerns**	56	46.3	23	39.7	3	30.0	2	13.3	33	50.0	47	43.5
	**No concerns**	14	11.6	9	15.5	1	10.0	2	13.3	8	12.1	14	13.0
	**I can not estimate**	0	0.0	1	1.7	1	10.0	1	6.7	0	0.0	1	0.9
	**Others (N/A)**	1	0.8	1	1.7	1	10.0	1	6.7	1	1.5	1	0.9
Interactions with other medication or food	**Major concerns**	19	15.7	8	13.8	2	20.0	5	33.3	13	19.7	11	10.2
	**Rather more concerns**	47	38.8	14	24.1	2	20.0	5	33.3	21	31.8	37	34.3
	**Rather less concerns**	45	37.2	30	51.7	3	30.0	4	26.7	24	36.4	50	46.3
	**No concerns**	9	7.4	4	6.9	2	20.0	0	0.0	7	10.6	8	7.4
	**I can not estimate**	0	0.0	1	1.7	0	0.0	0	0.0	0	0.0	1	0.9
	**Others (N/A)**	1	0.8	1	1.7	1	10.0	1	6.7	1	1.5	1	0.9

7. How helpful do you think the following sources of information are for finding out about self-medication products? Please only one answer option per line.

Participants replies - Main question follow by it's subcategories; broken down by gender and affiliation to a healthcare profession

		Gender (n =189)						Affiliation with a healthcare profession (n= 189)					
		Female (n = 121)		Male (= 58)		Non-binary or no specified (n=10)		Others (N/A) (n = 15)		Affiliated (fully trained & in training) (n=66)		No affiliation (n = 108)	
		absolut	%	absolut	%	absolut	%	absolut	%	absolut	%	absolut	%
Package leaflet	**Very helpful**	83	68.6	34	58.6	7	70.0	11	73.3	44	66.7	69	63.9
	**Rather helpful**	34	28.1	15	25.9	0	0.0	1	6.7	16	24.2	32	29.6
	**Less helpful**	3	2.5	8	13.8	2	20.0	2	13.3	6	9.1	5	4.6
	**Not helpful**	1	0.8	1	1.7	0	0.0	0	0.0	0	0.0	2	1.9
	**I can not estimate**	0	0.0	0	0.0	1	10.0	1	6.7	0	0.0	0	0.0
	**Others (N/A)**		0.0	0	0.0	0	0.0	0	0.0	0	0.0	0	0.0
Physicians	**Very helpful**	85	70.2	36	62.1	8	80.0	7	46.7	42	63.6	80	74.1
	**Rather helpful**	26	21.5	18	31.0	1	10.0	7	46.7	18	27.3	20	18.5
	**Less helpful**	8	6.6	4	6.9	0	0.0	0	0.0	5	7.6	7	6.5
	**Not helpful**	2	1.7	0	0.0	0	0.0	0	0.0	1	1.5	1	0.9
	**I can not estimate**	0	0.0	0	0.0	1	10.0	1	6.7	0	0.0	0	0.0
	**Others (N/A)**	0	0.0	0	0.0	0	0.0	0	0.0	0	0.0	0	0.0
Pharmacists	**Very helpful**	89	73.6	39	67.2	6	60.0	7	46.7	45	68.2	82	75.9
	**Rather helpful**	27	22.3	13	22.4	3	30.0	6	40.0	14	21.2	23	21.3
	**Less helpful**	1	0.8	4	6.9	0	0.0	1	6.7	3	4.5	1	0.9
	**Not helpful**	3	2.5	1	1.7	0	0.0	0	0.0	2	3.0	2	1.9
	**I can not estimate**	0	0.0	0	0.0	1	10.0	1	6.7	0	0.0	0	0.0
	**Others (N/A)**	1	0.8	1	1.7	0	0.0	0	0.0	2	3.0	0	0.0
Family member or friends	**Very helpful**	2	1.7	4	6.9	0	0.0	0	0.0	1	1.5	5	4.6
	**Rather helpful**	32	26.4	13	22.4	3	30.0	3	20.0	11	16.7	34	31.5
	**Less helpful**	62	51.2	27	46.6	5	50.0	7	46.7	36	54.5	51	47.2
	**Not helpful**	23	19.0	11	19.0	1	10.0	3	20.0	15	22.7	17	15.7
	**I can not estimate**	1	0.8	0	0.0	1	10.0	1	6.7	0	0.0	1	0.9
	**Others (N/A)**	1	0.8	3	5.2	0	0.0	1	6.7	3	4.5	0	0.0
Pharmacy magazines	**Very helpful**	1	0.8	1	1.7	0	0.0	1	6.7	0	0.0	1	0.9
	**Rather helpful**	22	18.2	8	13.8	3	30.0	1	6.7	11	16.7	21	19.4
	**Less helpful**	53	43.8	18	31.0	2	20.0	6	40.0	26	39.4	41	38.0
	**Not helpful**	29	24.0	21	36.2	3	30.0	5	33.3	23	34.8	25	23.1
	**I can not estimate**	16	13.2	9	15.5	2	20.0	2	13.3	5	7.6	20	18.5
	**Others (N/A)**	0	0.0	1	1.7	0	0.0	0	0.0	1	1.5	0	0.0
Health shows on TV/ radio	**Very helpful**	0	0.0	2	3.4	0	0.0	1	6.7	1	1.5	0	0.0
	**Rather helpful**	12	9.9	8	13.8	0	0.0	1	6.7	6	9.1	13	12.0
	**Less helpful**	37	30.6	10	17.2	1	10.0	2	13.3	16	24.2	30	27.8
	**Not helpful**	58	47.9	33	56.9	6	60.0	8	53.3	39	59.1	50	46.3
	**I can not estimate**	14	11.6	3	5.2	3	30.0	2	13.3	3	4.5	15	13.9
	**Others (N/A)**	0	0.0	2	3.4	0	0.0	1	6.7	1	1.5	0	0.0
Social media	**Very helpful**	2	1.7	0	0.0	0	0.0	0	0.0	0	0.0	2	1.9
	**Rather helpful**	5	4.1	2	3.4	0	0.0	1	6.7	0	0.0	6	5.6
	**Less helpful**	28	23.1	12	20.7	2	20.0	1	6.7	11	16.7	30	27.8
	**Not helpful**	71	58.7	39	67.2	6	60.0	11	73.3	47	71.2	58	53.7
	**I can not estimate**	15	12.4	2	3.4	2	20.0	1	6.7	6	9.1	12	11.1
	**Others (N/A)**	0	0.0	3	5.2	0	0.0	1	6.7	2	3.0	0	0.0
Manufacturers' website	**Very helpful**	8	6.6	8	13.8	2	20.0	1	6.7	8	12.1	9	8.3
	**Rather helpful**	46	38.0	8	13.8	2	20.0	1	6.7	24	36.4	31	28.7
	**Less helpful**	27	22.3	16	27.6	2	20.0	3	20.0	15	22.7	27	25.0
	**Not helpful**	28	23.1	19	32.8	3	30.0	8	53.3	17	25.8	25	23.1
	**I can not estimate**	11	9.1	6	10.3	1	10.0	2	13.3	1	1.5	15	13.9
	**Others (N/A)**	1	0.8	1	1.7	0	0.0	0	0.0	1	1.5	1	0.9
Health forums	**Very helpful**	3	2.5	1	1.7	1	10.0	0	0.0	2	3.0	3	2.8
	**Rather helpful**	32	26.4	17	29.3	4	40.0	5	33.3	18	27.3	30	27.8
	**Less helpful**	36	29.8	16	27.6	1	10.0	3	20.0	21	31.8	29	26.9
	**Not helpful**	31	25.6	17	29.3	2	20.0	6	40.0	20	30.3	24	22.2
	**I can not estimate**	19	15.7	6	10.3	2	20.0	1	6.7	4	6.1	22	20.4
	**Others (N/A)**	0	0.0	1	1.7	0	0.0	0	0.0	1	1.5	0	0.0
Websites of health insurance	**Very helpful**	2	1.7	2	3.4	1	10.0	0	0.0	3	4.5	2	1.9
	**Rather helpful**	35	28.9	18	31.0	0	0.0	6	40.0	12	18.2	35	32.4
	**Less helpful**	33	27.3	10	17.2	4	40.0	5	33.3	19	28.8	23	21.3
	**Not helpful**	29	24.0	17	29.3	1	10.0	3	20.0	22	33.3	22	20.4
	**I can not estimate**	22	18.2	10	17.2	4	40.0	1	6.7	9	13.6	26	24.1
	**Others (N/A)**	0	0.0	1	1.7	0	0.0	0	0.0	1	1.5	0	0.0
Mail-order pharmacies websites	**Very helpful**	2	1.7	1	1.7	0	0.0	0	0.0	0	0.0	3	2.8
	**Rather helpful**	22	18.2	10	17.2	0	0.0	0	0.0	11	16.7	21	19.4
	**Less helpful**	39	32.2	18	31.0	3	30.0	8	53.3	21	31.8	31	28.7
	**Not helpful**	34	28.1	20	34.5	2	20.0	5	33.3	24	36.4	27	25.0
	**I can not estimate**	23	19.0	8	13.8	5	50.0	2	13.3	9	13.6	25	23.1
	**Others (N/A)**	1	0.8	1	1.7	0	0.0	0	0.0	1	1.5	1	0.9
Website of physician's office	**Very helpful**	6	5.0	2	3.4	0	0.0	0	0.0	3	4.5	5	4.6
	**Rather helpful**	28	23.1	14	24.1	2	20.0	5	33.3	11	16.7	28	25.9
	**Less helpful**	30	24.8	15	25.9	1	10.0	5	33.3	17	25.8	24	22.2
	**Not helpful**	31	25.6	17	29.3	2	20.0	3	20.0	24	36.4	23	21.3
	**I can not estimate**	25	20.7	9	15.5	5	50.0	2	13.3	10	15.2	27	25.0
	**Others (N/A)**	1	0.8	1	1.7	0	0.0	0	0.0	1	1.5	1	0.9

8. How often do you inform yourself about the following aspects when using self-medication for the first time? Please only one answer option per line.

Participants replies - Main question follow by it's subcategories; broken down by gender and affiliation to a healthcare profession

		Gender (n =189)						Affiliation with a healthcare profession (n= 189)					
		Female (n = 121)		Male (= 58)		Non-binary or no specified (n=10)		Others (N/A) (n = 15)		Affiliated (fully trained & in training) (n=66)		No affiliation (n = 108)	
		absolut	%	absolut	%	absolut	%	absolut	%	absolut	%	absolut	%
Indication	**Always**	86	71.1	40	69.0	6	60.0	4	26.7	52	78.8	76	70.4
	**Mostly**	25	20.7	15	25.9	2	20.0	9	60.0	10	15.2	23	21.3
	**Rarely**	4	3.3	0	0.0	0	0.0	0	0.0	2	3.0	2	1.9
	**Never**	2	1.7	1	1.7	1	10.0	1	6.7	1	1.5	2	1.9
	**I can not estimate**	2	1.7	0	0.0	1	10.0	1	6.7	0	0.0	2	1.9
	**Others (N/A)**	2	1.7	2	3.4	0	0.0	0	0.0	1	1.5	3	2.8
Dosage	**Always**	94	77.7	50	86.2	7	70.0	9	60.0	55	83.3	87	80.6
	**Mostly**	18	14.9	6	10.3	2	20.0	5	33.3	8	12.1	13	12.0
	**Rarely**	2	1.7	0	0.0	0	0.0	0	0.0	0	0.0	2	1.9
	**Never**	2	1.7	1	1.7	1	10.0	1	6.7	1	1.5	2	1.9
	**I can not estimate**	1	0.8	0	0.0	0	0.0	0	0.0	0	0.0	1	0.9
	**Others (N/A)**	4	3.3	1	1.7	0	0.0	0	0.0	2	3.0	3	2.8
Special features in the administration	**Always**	65	53.7	31	53.4	7	70.0	8	53.3	36	54.5	59	54.6
	**Mostly**	44	36.4	17	29.3	2	20.0	4	26.7	23	34.8	36	33.3
	**Rarely**	5	4.1	6	10.3	0	0.0	2	13.3	4	6.1	5	4.6
	**Never**	4	3.3	3	5.2	1	10.0	1	6.7	2	3.0	5	4.6
	**I can not estimate**	1	0.8	0	0.0	0	0.0	0	0.0	0	0.0	1	0.9
	**Others (N/A)**	2	1.7	1	1.7	0	0.0	0	0.0	1	1.5	2	1.9
Possible adverse drug effect	**Always**	44	36.4	19	32.8	6	60.0	6	40.0	26	39.4	37	34.3
	**Mostly**	41	33.9	22	37.9	1	10.0	7	46.7	19	28.8	38	35.2
	**Rarely**	29	24.0	11	19.0	2	20.0	1	6.7	17	25.8	24	22.2
	**Never**	2	1.7	4	6.9	1	10.0	1	6.7	1	1.5	5	4.6
	**I can not estimate**	3	2.5	0	0.0	0	0.0	0	0.0	1	1.5	2	1.9
	**Others (N/A)**	2	1.7	2	3.4	0	0.0	0	0.0	2	3.0	2	1.9
Interactions	**Always**	52	43.0	15	25.9	6	60.0	6	40.0	27	40.9	40	37.0
	**Mostly**	26	21.5	19	32.8	2	20.0	4	26.7	15	22.7	28	25.9
	**Rarely**	34	28.1	19	32.8	1	10.0	4	26.7	19	28.8	31	28.7
	**Never**	5	4.1	4	6.9	1	10.0	1	6.7	4	6.1	5	4.6
	**I can not estimate**	2	1.7	0	0.0	0	0.0	0	0.0	0	0.0	2	1.9
	**Others (N/A)**	2	1.7	1	1.7	0	0.0	0	0.0	1	1.5	2	1.9
Maximum duration of use without medical advice	**Always**	48	39.7	20	34.5	4	40.0	5	33.3	32	48.5	35	32.4
	**Mostly**	40	33.1	22	37.9	3	30.0	7	46.7	17	25.8	41	38.0
	**Rarely**	23	19.0	8	13.8	2	20.0	2	13.3	10	15.2	21	19.4
	**Never**	4	3.3	6	10.3	1	10.0	1	6.7	6	9.1	4	3.7
	**I can not estimate**	4	3.3	1	1.7	0	0.0	0	0.0	0	0.0	5	4.6
	**Others (N/A)**	2	1.7	1	1.7	0	0.0	0	0.0	1	1.5	2	1.9
Possible contraindication	**Always**	65	53.7	16	27.6	8	80.0	8	53.3	31	47.0	50	46.3
	**Mostly**	30	24.8	22	37.9	1	10.0	5	33.3	20	30.3	28	25.9
	**Rarely**	17	14.0	11	19.0	0	0.0	1	6.7	10	15.2	17	15.7
	**Never**	4	3.3	7	12.1	1	10.0	1	6.7	4	6.1	7	6.5
	**I can not estimate**	3	2.5	1	1.7	0	0.0	0	0.0	0	0.0	4	3.7
	**Others (N/A)**	2	1.7	1	1.7	0	0.0	0	0.0	1	1.5	2	1.9

9. How important are the following factors to you when deciding to self-medicate? Please only one answer option per line.

Participants replies - Main question follow by it's subcategories; broken down by gender and affiliation to a healthcare profession

		Gender (n =189)						Affiliation with a healthcare profession (n= 189)					
		Female (n = 121)		Male (= 58)		Non-binary or no specified (n=10)		Others (N/A) (n = 15)		Affiliated (fully trained & in training) (n=66)		No affiliation (n = 108)	
		absolut	%	absolut	%	absolut	%	absolut	%	absolut	%	absolut	%
Physician’s recommendation	**Very important**	74	61.2	32	55.2	6	60.0	7	46.7	34	51.5	71	65.7
	**Rather important**	36	29.8	20	34.5	1	10.0	5	33.3	26	39.4	26	24.1
	**Less important**	5	4.1	5	8.6	3	30.0	2	13.3	2	3.0	9	8.3
	**Not Important**	3	2.5	1	1.7	0	0.0	1	6.7	3	4.5	0	0.0
	**I can not estimate**	1	0.8	0	0.0	0	0.0	0	0.0	0	0.0	1	0.9
	**Others (N/A)**	2	1.7	0	0.0	0	0.0	0	0.0	1	1.5	1	0.9
Pharmacist’s recommendation	**Very important**	60	49.6	27	46.6	5	50.0	7	46.7	30	45.5	55	50.9
	**Rather important**	49	40.5	21	36.2	3	30.0	6	40.0	26	39.4	41	38.0
	**Less important**	6	5.0	7	12.1	2	20.0	2	13.3	7	10.6	6	5.6
	**Not Important**	4	3.3	3	5.2	0	0.0	0	0.0	3	4.5	4	3.7
	**I can not estimate**	1	0.8	0	0.0	0	0.0	0	0.0	0	0.0	1	0.9
	**Others (N/A)**	1	0.8	0	0.0	0	0.0	0	0.0	0	0.0	1	0.9
Recommendation by family members or partners	**Very important**	7	5.8	4	6.9	0	0.0	1	6.7	1	1.5	9	8.3
	**Rather important**	39	32.2	18	31.0	4	40.0	6	40.0	19	28.8	36	33.3
	**Less important**	53	43.8	25	43.1	4	40.0	4	26.7	33	50.0	45	41.7
	**Not Important**	20	16.5	10	17.2	2	20.0	4	26.7	13	19.7	15	13.9
	**I can not estimate**	1	0.8	1	1.7	0	0.0	0	0.0	0	0.0	2	1.9
	**Others (N/A)**	1	0.8	0	0.0	0	0.0	0	0.0	0	0.0	1	0.9
Recommendation from health shows on TV/ radio	**Very important**	1	0.8	3	5.2	0	0.0	1	6.7	2	3.0	1	0.9
	**Rather important**	10	8.3	6	10.3	1	10.0	0	0.0	4	6.1	13	12.0
	**Less important**	41	33.9	15	25.9	3	30.0	6	40.0	16	24.2	37	34.3
	**Not Important**	62	51.2	33	56.9	6	60.0	8	53.3	42	63.6	51	47.2
	**I can not estimate**	6	5.0	1	1.7	0	0.0	0	0.0	2	3.0	5	4.6
	**Others (N/A)**	1	0.8	0	0.0	0	0.0	0	0.0	0	0.0	1	0.9
Good experience with medication	**Very important**	55	45.5	18	31.0	5	50.0	7	46.7	26	39.4	45	41.7
	**Rather important**	54	44.6	32	55.2	2	20.0	6	40.0	32	48.5	50	46.3
	**Less important**	5	4.1	5	8.6	2	20.0	0	0.0	5	7.6	7	6.5
	**Not Important**	4	3.3	2	3.4	1	10.0	2	13.3	2	3.0	3	2.8
	**I can not estimate**	1	0.8	1	1.7	0	0.0	0	0.0	0	0.0	2	1.9
	**Others (N/A)**	2	1.7	0	0.0	0	0.0	0	0.0	1	1.5	1	0.9
Price	**Very important**	6	5.0	5	8.6	4	40.0	3	20.0	2	3.0	10	9.3
	**Rather important**	29	24.0	14	24.1	3	30.0	6	40.0	18	27.3	22	20.4
	**Less important**	49	40.5	20	34.5	0	0.0	2	13.3	25	37.9	42	38.9
	**Not Important**	30	24.8	17	29.3	2	20.0	4	26.7	18	27.3	27	25.0
	**I can not estimate**	6	5.0	2	3.4	0	0.0	0	0.0	2	3.0	6	5.6
	**Others (N/A)**	1	0.8	0	0.0	1	10.0	0	0.0	1	1.5	1	0.9
Duration until medication is available	**Very important**	14	11.6	7	12.1	3	30.0	2	13.3	8	12.1	14	13.0
	**Rather important**	46	38.0	17	29.3	1	10.0	3	20.0	24	36.4	37	34.3
	**Less important**	27	22.3	16	27.6	1	10.0	2	13.3	11	16.7	31	28.7
	**Not Important**	18	14.9	15	25.9	3	30.0	7	46.7	14	21.2	15	13.9
	**I can not estimate**	14	11.6	3	5.2	2	20.0	1	6.7	9	13.6	9	8.3
	**Others (N/A)**	2	1.7	0	0.0	0	0.0	0	0.0	0	0.0	2	1.9
Packing appearance	**Very important**	0	0.0	0	0.0	0	0.0	0	0.0	0	0.0	0	0.0
	**Rather important**	2	1.7	1	1.7	1	10.0	1	6.7	0	0.0	3	2.8
	**Less important**	12	9.9	6	10.3	0	0.0	1	6.7	6	9.1	11	10.2
	**Not Important**	98	81.0	50	86.2	7	70.0	12	80.0	59	89.4	84	77.8
	**I can not estimate**	8	6.6	1	1.7	2	20.0	1	6.7	1	1.5	9	8.3
	**Others (N/A)**	1	0.8	0	0.0	0	0.0	0	0.0	0	0.0	1	0.9
TV-commercial	**Very important**	0	0.0	0	0.0	0	0.0	0	0.0	0	0.0	0	0.0
	**Rather important**	0	0.0	1	1.7	0	0.0	1	6.7	0	0.0	0	0.0
	**Less important**	11	9.1	5	8.6	0	0.0	1	6.7	5	7.6	10	9.3
	**Not Important**	101	83.5	51	87.9	10	100.0	13	86.7	61	92.4	88	81.5
	**I can not estimate**	8	6.6	0	0.0	0	0.0	0	0.0	0	0.0	8	7.4
	**Others (N/A)**	1	0.8	1	1.7	0	0.0	0	0.0	0	0.0	2	1.9
Advertising in magazines	**Very important**	0	0.0	1	1.7	0	0.0	1	6.7	0	0.0	0	0.0
	**Rather important**	0	0.0	0	0.0	0	0.0	0	0.0	0	0.0	0	0.0
	**Less important**	9	7.4	4	6.9	10	100.0	2	13.3	3	4.5	8	7.4
	**Not Important**	103	85.1	53	91.4	0	0.0	12	80.0	63	95.5	91	84.3
	**I can not estimate**	8	6.6	0	0.0	0	0.0	0	0.0	0	0.0	8	7.4
	**Others (N/A)**	1	0.8	0	0.0	0	0.0	0	0.0	0	0.0	1	0.9
Advertisement on internet sites	**Very important**	0	0.0	0	0.0	0	0.0	0	0.0	0	0.0	0	0.0
	**Rather important**	2	1.7	1	1.7	0	0.0	1	6.7	0	0.0	2	1.9
	**Less important**	15	12.4	4	6.9	10	100.0	2	13.3	5	7.6	12	11.1
	**Not Important**	96	79.3	53	91.4	0	0.0	12	80.0	61	92.4	86	79.6
	**I can not estimate**	7	5.8	0	0.0	0	0.0	0	0.0	0	0.0	7	6.5
	**Others (N/A)**	1	0.8	0	0.0	0	0.0	0	0.0	0	0.0	1	0.9
Recommendation on social media	**Very important**	0	0.0	1	1.7	0	0.0	0	0.0	0	0.0	1	0.9
	**Rather important**	4	3.3	0	0.0	0	0.0	0	0.0	0	0.0	4	3.7
	**Less important**	13	10.7	8	13.8	2	20.0	5	33.3	1	1.5	17	15.7
	**Not Important**	98	81.0	48	82.8	8	80.0	10	66.7	64	97.0	80	74.1
	**I can not estimate**	5	4.1	1	1.7	0	0.0	0	0.0	1	1.5	5	4.6
	**Others (N/A)**	1	0.8	0	0.0	0	0.0	0	0.0	0	0.0	1	0.9

**10. How much do the following factors influence your decision to self-medicate before seeing a physician?** Please only one answer option per line.		Gender (n =189)						Affiliation with a healthcare profession (n= 189)					
		Female (n = 121)		Male (= 58)		Non-binary or no specified (n=10)		Others (N/A) (n = 15)		Affiliated (fully trained & in training) (n=66)		No affiliation (n = 108)	
		absolut	%	absolut	%	absolut	%	absolut	%	absolut	%	absolut	%
Participants replies - Main question follow by it's subcategories; broken down by gender and affiliation to a healthcare profession													
Intensity of complaints	**Very strong**	74	61.2	37	63.8	5	50.0	8	53.3	40	60.6	68	63.0
	**Strong**	39	32.2	18	31.0	2	20.0	5	33.3	24	36.4	30	27.8
	**Less strong**	1	0.8	3	5.2	2	20.0	0	0.0	2	3.0	4	3.7
	**Not at all**	4	3.3	0	0.0	1	10.0	2	13.3	0	0.0	3	2.8
	**I can not estimate**	3	2.5	0	0.0	0	0.0	0	0.0	0	0.0	3	2.8
	**Others (N/A)**	0	0.0	0	0.0	0	0.0	0	0.0	0	0.0	0	0.0
Duration of the complaints	**Very strong**	60	49.6	28	48.3	4	40.0	8	53.3	35	53.0	49	45.4
	**Strong**	43	35.5	23	39.7	2	20.0	4	26.7	26	39.4	38	35.2
	**Less strong**	7	5.8	2	3.4	2	20.0	0	0.0	2	3.0	9	8.3
	**Not at all**	6	5.0	3	5.2	1	10.0	2	13.3	2	3.0	6	5.6
	**I can not estimate**	3	2.5	2	3.4	0	0.0	0	0.0	1	1.5	4	3.7
	**Others (N/A)**	2	1.7	0	0.0	1	10.0	1	6.7	0	0.0	2	1.9
Experience with the complaints	**Very strong**	66	54.5	25	43.1	6	60.0	7	46.7	34	51.5	56	51.9
	**Strong**	42	34.7	30	51.7	2	20.0	6	40.0	26	39.4	42	38.9
	**Less strong**	8	6.6	2	3.4	0	0.0	0	0.0	2	3.0	8	7.4
	**Not at all**	2	1.7	0	0.0	1	10.0	2	13.3	0	0.0	1	0.9
	**I can not estimate**	0	0.0	1	1.7	1	10.0	0	0.0	2	3.0	0	0.0
	**Others (N/A)**	3	2.5	0	0.0	0	0.0	0	0.0	2	3.0	1	0.9
Advice of family or partners	**Very strong**	13	10.7	5	8.6	2	20.0	0	0.0	6	9.1	14	13.0
	**Strong**	37	30.6	18	31.0	4	40.0	8	53.3	15	22.7	36	33.3
	**Less strong**	50	41.3	25	43.1	0	0.0	2	13.3	30	45.5	43	39.8
	**Not at all**	18	14.9	9	15.5	3	30.0	3	20.0	13	19.7	14	13.0
	**I can not estimate**	0	0.0	1	1.7	0	0.0	0	0.0	1	1.5	0	0.0
	**Others (N/A)**	3	2.5	0	0.0	1	10.0	2	13.3	1	1.5	1	0.9
Advice from friends	**Very strong**	8	6.6	3	5.2	1	10.0	1	6.7	3	4.5	8	7.4
	**Strong**	24	19.8	13	22.4	3	30.0	3	20.0	14	21.2	23	21.3
	**Less strong**	61	50.4	27	46.6	2	20.0	6	40.0	32	48.5	52	48.1
	**Not at all**	25	20.7	12	20.7	3	30.0	3	20.0	15	22.7	22	20.4
	**I can not estimate**	1	0.8	2	3.4	0	0.0	0	0.0	1	1.5	2	1.9
	**Others (N/A)**	2	1.7	1	1.7	1	10.0	2	13.3	1	1.5	1	0.9
Own research on the complaints	**Very strong**	9	7.4	11	19.0	1	10.0	2	13.3	11	16.7	8	7.4
	**Strong**	27	22.3	11	19.0	2	20.0	1	6.7	7	10.6	32	29.6
	**Less strong**	47	38.8	18	31.0	1	10.0	3	20.0	31	47.0	32	29.6
	**Not at all**	34	28.1	16	27.6	5	50.0	8	53.3	14	21.2	33	30.6
	**I can not estimate**	3	2.5	1	1.7	0	0.0	0	0.0	3	4.5	1	0.9
	**Others (N/A)**	1	0.8	1	1.7	1	10.0	1	6.7	0	0.0	2	1.9
Availability of physician's appointment	**Very strong**	20	16.5	11	19.0	3	30.0	4	26.7	10	15.2	20	18.5
	**Strong**	49	40.5	19	32.8	4	40.0	3	20.0	30	45.5	39	36.1
	**Less strong**	37	30.6	17	29.3	0	0.0	4	26.7	20	30.3	30	27.8
	**Not at all**	10	8.3	9	15.5	1	10.0	3	20.0	4	6.1	23	21.3
	**I can not estimate**	4	3.3	2	3.4	1	10.0	0	0.0	2	3.0	5	4.6
	**Others (N/A)**	1	0.8	0	0.0	1	10.0	1	6.7	0	0.0	1	0.9
Trust in my physician	**Very strong**	21	17.4	18	31.0	2	20.0	2	13.3	14	21.2	25	23.1
	**Strong**	49	40.5	13	22.4	1	10.0	6	40.0	25	37.9	32	29.6
	**Less strong**	23	19.0	8	13.8	1	10.0	2	13.3	10	15.2	20	18.5
	**Not at all**	22	18.2	15	25.9	3	30.0	4	26.7	13	19.7	23	21.3
	**I can not estimate**	4	3.3	4	6.9	2	20.0	0	0.0	4	6.1	6	5.6
	**Others (N/A)**	2	1.7	0	0.0	1	10.0	1	6.7	0	0.0	2	1.9
